# Comprehensive plasma proteomic profiling reveals biomarkers for active tuberculosis

**DOI:** 10.1172/jci.insight.137427

**Published:** 2020-09-17

**Authors:** Diana J. Garay-Baquero, Cory H. White, Naomi F. Walker, Marc Tebruegge, Hannah F. Schiff, Cesar Ugarte-Gil, Stephen Morris-Jones, Ben G. Marshall, Antigoni Manousopoulou, John Adamson, Andres F. Vallejo, Magdalena K. Bielecka, Robert J. Wilkinson, Liku B. Tezera, Christopher H. Woelk, Spiros D. Garbis, Paul Elkington

**Affiliations:** 1 School of Clinical and Experimental Sciences, Faculty of Medicine, and; 2 Institute for Life Sciences, University of Southampton, Southampton, United Kingdom.; 3 Proteome Exploration Laboratory, Beckman Institute, California Institute of Technology, Pasadena, California, USA.; 4 Wellcome Centre for Infectious Diseases Research in Africa, Institute of Infectious Disease and Molecular Medicine, University of Cape Town, Observatory 7925, South Africa.; 5 Department of Clinical Sciences, Liverpool School of Tropical Medicine, Liverpool, United Kingdom.; 6 Department of Medicine, University of Cape Town, Observatory 7925, South Africa.; 7 TB Centre and Department of Clinical Research, London School of Hygiene & Tropical Medicine, London, United Kingdom.; 8 Department of Paediatric Infectious Diseases & Immunology, Evelina London Children’s Hospital, Guy’s and St Thomas’ NHS Foundation Trust, London, United Kingdom.; 9 Department of Infection, Immunity and Inflammation, UCL Great Ormond Street Institute of Child Health, University College London, London, United Kingdom.; 10 Department of Paediatrics, University of Melbourne, Parkville, Australia.; 11 Instituto de Medicina Tropical Alexander von Humboldt, School of Medicine, Universidad Peruana Cayetano Heredia, Lima, Peru.; 12 Department of Microbiology, University College London Hospitals NHS Trust, London, United Kingdom.; 13 Division of Infection and Immunity, University College London, London, United Kingdom.; 14 National Institute for Health Research (NIHR) Biomedical Research Centre, University Hospital NHS Foundation Trust, Southampton, Southampton, United Kingdom.; 15 Pharmacology Core, Africa Health Research Institute (AHRI), Durban, South Africa.; 16 The Francis Crick Institute, London, United Kingdom.; 17 Department of Infectious Diseases, Faculty of Medicine, Imperial College, London, United Kingdom.; 18 Cancer Sciences Division, Faculty of Medicine, University of Southampton, United Kingdom.

**Keywords:** Infectious disease, Diagnostics, Proteomics, Tuberculosis

## Abstract

**BACKGROUND:**

Tuberculosis (TB) kills more people than any other infection, and new diagnostic tests to identify active cases are required. We aimed to discover and verify novel markers for TB in nondepleted plasma.

**METHODS:**

We applied an optimized quantitative proteomics discovery methodology based on multidimensional and orthogonal liquid chromatographic separation combined with high-resolution mass spectrometry to study nondepleted plasma of 11 patients with active TB compared with 10 healthy controls. Prioritized candidates were verified in independent UK (*n* = 118) and South African cohorts (*n* = 203).

**RESULTS:**

We generated the most comprehensive TB plasma proteome to date, profiling 5022 proteins spanning 11 orders-of-magnitude concentration range with diverse biochemical and molecular properties. We analyzed the predominantly low–molecular weight subproteome, identifying 46 proteins with significantly increased and 90 with decreased abundance (peptide FDR ≤ 1%, *q* ≤ 0.05). Verification was performed for novel candidate biomarkers (CFHR5, ILF2) in 2 independent cohorts. Receiver operating characteristics analyses using a 5-protein panel (CFHR5, LRG1, CRP, LBP, and SAA1) exhibited discriminatory power in distinguishing TB from other respiratory diseases (AUC = 0.81).

**CONCLUSION:**

We report the most comprehensive TB plasma proteome to date, identifying novel markers with verification in 2 independent cohorts, leading to a 5-protein biosignature with potential to improve TB diagnosis. With further development, these biomarkers have potential as a diagnostic triage test.

**FUNDING:**

Colciencias, Medical Research Council, Innovate UK, NIHR, Academy of Medical Sciences, Program for Advanced Research Capacities for AIDS, Wellcome Centre for Infectious Diseases Research.

## Abstract 2

Analysis of a comprehensive tuberculosis plasma proteome identified a 5-protein biosignature with potential to improve active tuberculosis diagnosis.

## Introduction

The tuberculosis (TB) pandemic continues relentlessly, killing more humans than any other infectious disease, and progress in its containment is lagging behind other major diseases, such as HIV and malaria (1). A fundamental issue with controlling the global pandemic is the inadequacy of current diagnostic tests for TB, which have multiple limitations, such as insufficient sensitivity, high cost, and reliance on laboratory infrastructure (2, 3). The World Health Organization (WHO) has defined the characteristics of an optimal TB diagnostic, including low cost, use of a nonsputum sample, high sensitivity and specificity, as well as stability at extremes of temperature and humidity, and it may include both rule-in and rule-out tests (4). However, development of a point-of-care test suitable for resource-limited settings faces multiple challenges in the pathway from discovery to validation and implementation, such as translation between platforms, application across different populations, and the disease heterogeneity of TB.

Proteins have been proposed as viable diagnostic candidates given their phenotypic relevance and stability under specified conditions. Blood plasma contains a wide spectrum of proteins that may serve as biological signatures of physiological status during homeostasis or its perturbation (5). For example, the plasma matrix encompasses tissue leakage proteins, thus providing systemic and organotypic insight about specific immunopathologic features, such as lung tissue destruction, relevant to active TB (6, 7). Furthermore, plasma protein signatures are highly amenable for translation to rapid test devices, and this technology is rapidly evolving, including colorimetric gold nanoparticles on paper-based devices, label-free biosensors, and nanofluidic disposable chips (8, 9). Extensive proteomic discovery research has been conducted in TB. Although this has identified novel diagnostic markers for the active disease (10–16) and progression from latent disease (17), an optimal diagnostic panel has yet to be defined (18). Other analytes, such as matrix degradation products, have been found by a hypothesis-driven approach (7, 19) but conversely have not been identified by mass spectrometry–based strategies. This implies that improved discovery strategies are required to increase the plasma proteome coverage, thus improving the prospect of capturing novel protein markers with potential clinical utility.

**Figure 1:**
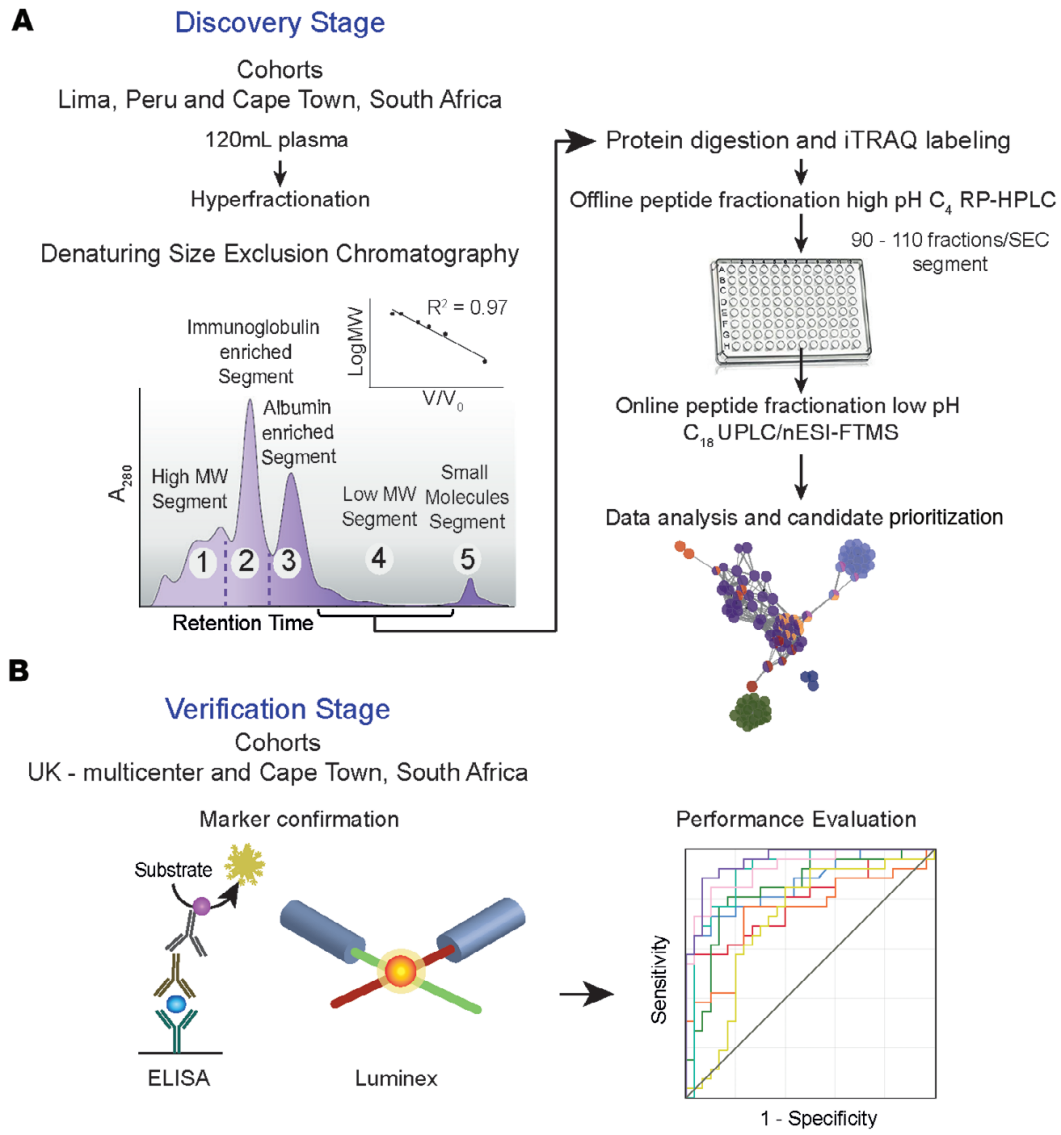
Overview of the plasma proteomic discovery and validation strategy of potential TB biomarkers. (**A**) Identification and quantification of plasma proteins were performed using a quantitative multidimensional protein identification approach, which comprises a series of fractionation steps at both protein (denaturing high-performance–size exclusion chromatography, HP-SEC) and peptide levels (offline high pH C4 HPLC followed by online low pH C18 ultrahigh-performance LC, UPLC). Initial plasma prefractionation using HP-SEC produces 5 segments depending on the molecular size. Only segments 1 to 4 were included in this study because these included most of the protein contents. (**B**) Bioinformatic processing prioritized markers, which were then measured by ELISA or Luminex in plasma or serum samples from 2 cohorts. Discovery and validation stages involved multiple ethnicities. iTRAQ, isobaric tags for relative and absolute quantitation; nESI-FTMS, nano-electrospray ionization Fourier-transform mass spectrometry.

Current limitations to mainstream serum or plasma proteomics pipelines partly stem from the predominance in protein mass (>95%) of the top 20 most abundant proteins. These high-abundance proteins either mask the presence of or are noncovalently bound to lower abundance proteins with potential clinical relevance. In an effort to overcome this limitation, an initial serum/plasma depletion step to remove such high-abundance proteins is typically employed before the mass spectrometry–based analysis. This plasma proteome analysis strategy has been used in samples from patients with TB (11, 13, 20–24). However, this approach will result in the inadvertent loss of a wide spectrum of physiologically important proteins, including those typically encountered in lipid microvesicles, such as exosomes, proteases and their cleavage products, and native peptides such as hormones (25, 26). Consequently, an alternative methodological approach has been optimized, wherein the entire repertoire of secreted and exosome-enriched proteins, including the high-abundance carrier and immunoglobulin proteins, and their derivative proteotypic peptides are subjected to multidimensional or orthogonal liquid chromatographic separation combined with high-definition mass spectrometry analysis (Figure 1) (27–29). The present study optimized critical aspects of this methodology to generate a highly comprehensive plasma proteome coverage to capture potentially novel biomarkers in active TB.

## Results

### Proteomic analysis of nondepleted plasma identifies numerous modulated proteins in TB.

**Figure 2:**
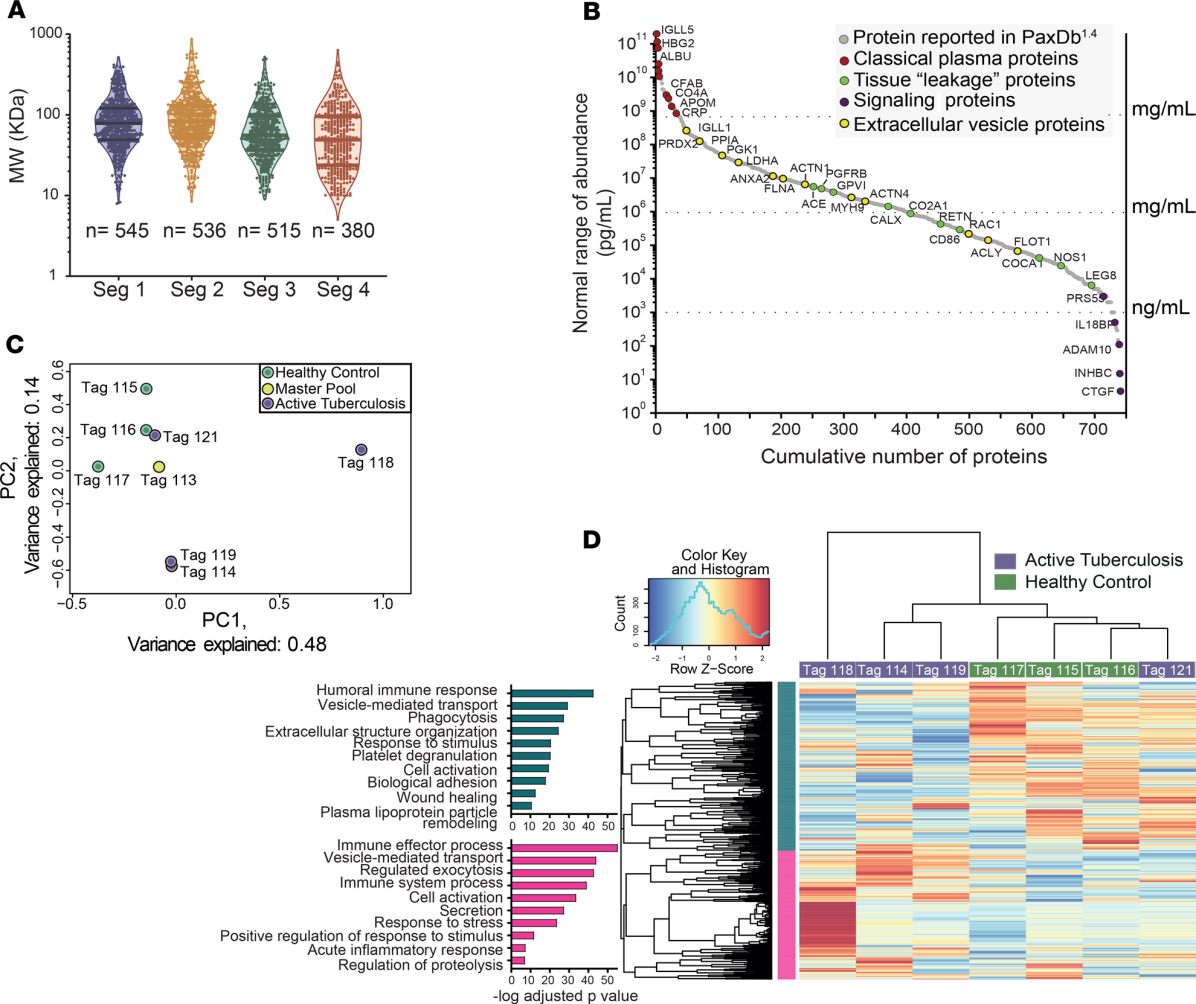
In-depth quantitative plasma proteome profiling in TB. (**A**) Violin plots with median and interquartile range show molecular weight frequency distributions of proteins quantified (peptide confidence ≤ 1% FDR) in each independent HP-SEC segment. The number of proteins with relative quantitative data in all profiled samples is indicated. Four plasma samples from TB patients, 3 healthy controls, and 1 master pool were analyzed. (**B**) Abundance of quantified proteins from all HP-SEC segments. Only proteins with circulating levels reported in the reference PaxDb^4.1^ protein abundance database or in the literature were annotated. Proteins considered as classical plasma proteins are indicated in red, tissue leakage proteins in green, proteins with signaling functions in purple, and proteins associated with extracellular vesicles in yellow. Concentrations of detected proteins span 11 orders of magnitude. (**C**) Principal components analysis (PCA) based on quantified proteins from all HP-SEC segments of 8 profiled samples. iTRAQ tags and groups are indicated. Overall, TB patients were separated from healthy controls by the principal components PC1 and PC2, collectively explaining the 62% of total variance. The TB sample labeled with tag 121 clustered with the healthy control samples. The master pool, a combination of all samples, was located in the center of the samples. (**D**) Log_2_-transformed relative protein expression heatmap of all proteins profiled in the 4 HP-SEC segments. Purple indicates TB patients and green healthy controls. Pearson correlation was used for clustering of proteins and Spearman’s for samples. Two clusters were defined based on the relative protein expression, and Gene Ontology (GO) analysis of these was performed using g:Profiler. Cyan, downregulated proteins; magenta, upregulated proteins.

For each sample, 4 protein segments were generated from plasma by HP-SEC partitioning under highly chaotropic mobile phase conditions. Then, each HP-SEC segment was subjected to downstream 2D LC tandem mass spectrometry (LC-MS) analysis to achieve a comprehensive profile of the nondepleted plasma proteome (Supplemental Figure 1; supplemental material available online with this article; https://doi.org/10.1172/jci.insight.137427DS1). The HP-SEC fractionation traces were highly reproducible (Supplemental Figure 2). All 4 segments from 1 set of 7 plasma samples, comprising 4 samples from active TB patients and 3 from healthy donors, and 1 master pool (Set A, Supplemental Table 1) were profiled to generate an exploratory in-depth plasma proteome in TB (Figure 2). Samples included in this first stage were obtained from donors from South Africa and Peru. The samples from Peru were collected prospectively to match BMI and age of the donors from South Africa (Supplemental Table 1). A total of 5022 nonredundant proteins (peptide FDR ≤ 5%) were identified, from which 3577 were quantified across all 8 samples. Only quantified proteins profiled at a strict 1% FDR were subjected to further bioinformatic and statistical analysis. Proteins profiled in the subproteome contained in segment 4 presented the widest distribution of molecular weight, ranging from 5 kDa to 630 kDa (Figure 2A). A total of 53% of the quantified proteins had reported circulating levels in the literature or the human plasma data set (integrated) from the reference PaxDb^4.1^ protein abundance database (30, 31). Based on these reported circulating levels, the plasma proteomic profile covered abundance levels of 11 orders of magnitude (Figure 2B), representing classical, tissue leakage, and signaling proteins (32). Furthermore, 905 profiled proteins were annotated as exosome-, microvesicle-, or microparticle-derived proteins (33). The actual abundance dynamic range is expected to be larger, as the LC-MS signal intensity observed for many proteins with unknown native concentration levels was below that of proteins with the lowest reported concentrations (30).

PCA demonstrated that this plasma proteome could distinguish between controls and patients with active TB (Figure 2C). Overall, 62% of the variance was explained by PC1 and PC2. The master pool was a combination of plasma from healthy control and TB patients and clustered in the center of control and diseased groups. One TB patient profile (reporter ion at *m/z* 121) clustered with the control group, and review of the clinical data showed that although the *Mycobacterium tuberculosis* (*M. tuberculosis*) sputum culture was positive, the plasma C-reactive protein (CRP) level was normal and the chest x-ray showed no consolidation, suggesting very early disease, in contrast to all other patients who had lung inflammation. This demonstrates that proteomic profiling reflects disease heterogeneity that is consistent with clinical features.

Similar to the PCA, Spearman’s correlation showed clustering between TB and controls but with reporter ion at *m/z* 121 clustering with controls (Figure 2D). Defined patterns of protein expression associated with the disease status were observed in 2 clusters. Cluster blue includes proteins with reduced abundance in the TB group while cluster magenta contains proteins with increased abundance in the TB group. Gene ontology enrichment analysis indicated regulation of immune response to external stimulus mainly through the innate response, including the complement pathway and phagocytosis.

Recently, analytical models such as Linear Models for Microarray Data (limma) have been translated to proteomic data sets from large-scale gene expression data (34). Empirical Bayes approaches have been proven to be particularly powerful with small sample numbers by using the full data sets to reduce observed sample variances toward an estimate while allowing for variance distribution (35–37). This statistical approach results in a more realistic distribution of biological variances compared with other methods. Furthermore, limma offered the best statistical properties when compared with generalized linear model and mixed models in the context of multiplexed isobaric quantitative proteomics (34). Statistical assessment of differential expression showed 119 proteins were significantly modulated (nominal *P* ≤ 0.05) (Supplemental Table 2). However, after FDR correction for multiple comparisons, no significant differences were retained. Therefore, we increased the sample size to identify TB biomarker proteins with high confidence.

### In-depth analysis of segment 4 identifies multiple new TB biomarkers.

Robust statistics are crucial at the discovery stage of biomarker identification to increase chances of later validation. Considering that HP-SEC segment 4 captured the most diverse range of protein molecular weight (Figure 2A), we interrogated this segment further to increase statistical power. Reported simulations for statistical power in proteomic studies, including power curves estimated for iTRAQ relative ratios (37), predict that a minimum of 9 biological or clinical replicate samples per group are needed to achieve a statistical power of 0.9 when an effect size of 1.5 is considered (37, 38). Therefore, 10 healthy control and 11 active TB plasma samples were analyzed. These samples were randomly allocated into 3 iTRAQ experiments (Supplemental Figure 3A) and analyzed as 3 independent MS experiments. A maximum of 1248 proteins were quantified at 1% FDR, and 426 proteins were common to the 3 MS runs (Supplemental Figure 3B). The overall relative protein expression variation was evaluated using the common proteins profiled across the 3 independent iTRAQ experiments. The relative standard deviation (RSD) was more than 25, which accounts for the combined technical and biological variation (Supplemental Figure 3C). Using an alternative approach to estimate the mean-variance relationship in the data, the locally weighted regression (LOWESS) trend was calculated using the function voom (39) from the limma R package, analyzing the same group of proteins (Supplemental Figure 3D). The square-root-standard-deviation, sqrt(SD), was more than 1.4, and the LOWESS voom trend indicated a degree of heteroscedasticity in the data, where greater log_2_ relative expression values were related to higher variation. The range of RSD and sqrt(SD) estimated across these 3 multidimensional experiments indicated a good overall method performance.

**Figure 3:**
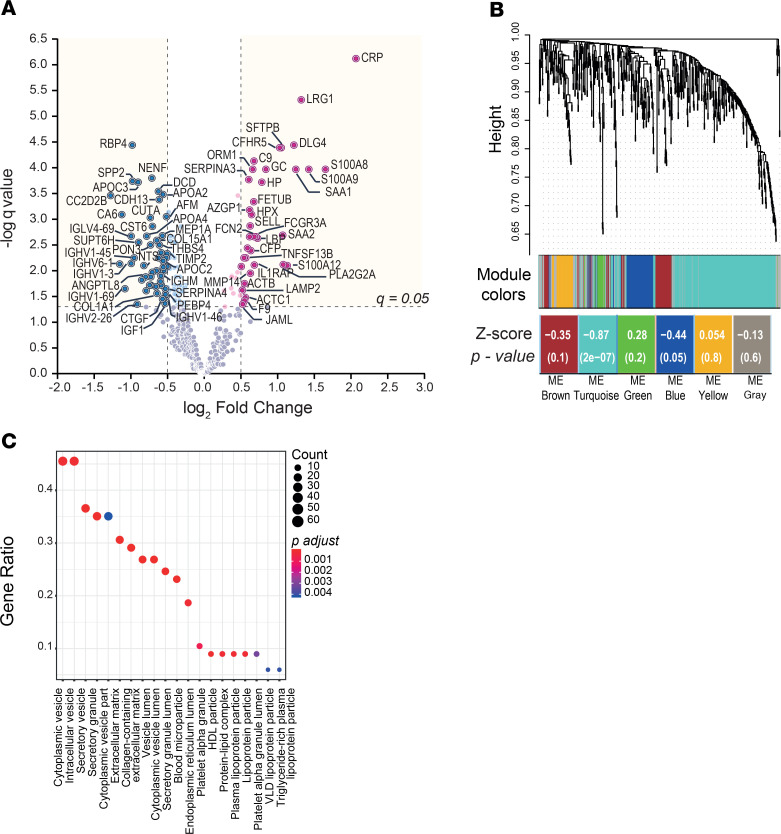
Detailed profiling of segment 4 identifies a differential plasma proteome in TB infection. Analyses of common quantified proteins (peptide confidence FDR ≤ 1%) derived from HP-SEC segment 4 across 3 iTRAQ experiments studying 10 controls and 11 TB patients (*n* = 426 proteins). (**A**) Volcano plot representation of plasma proteins differentially expressed in TB defined by limma with FDR correction (*q* ≤ 0.05). Pink indicates upregulated proteins and blue downregulated. Gene names of significantly regulated proteins with log_2_ fold change ≥ |0.5| are shown. (**B**) WGCNA cluster dendrogram of quantified proteins into distinctive modules defined by dendogram branch cutting. Color modules indicate protein clusters of highly interconnected proteins associated with the disease status. Correlation score and significance demonstrates that module turquoise is strongly correlated to TB status. ME, Module Eigengenes. (**C**) GO enrichment of proteins included in the module turquoise (*n* = 189). Dots represent the top 20 enriched cellular component organization terms. Dot color indicates significance (*P* value Benjamini-Hochberg adjusted), and size represents the number of differential proteins in the significant gene list associated with the GO term.

The data sets generated were inspected to evaluate batch effects and data distribution. Sixty percent of the variance was explained by the batch (Supplemental Figure 4A). The group effect was then distinguishable when considering dimensions PC2 and PC3 (~17% variance, Supplemental Figure 4B). Batch effect correction was performed using normalization to the master pool or by ComBat (40) (Supplemental Figure 4, C and D, respectively), with ComBat providing the best reduction of batch effects. Statistical assessment of significant differential protein expression using limma revealed 136 proteins significantly modulated (*q* ≤ 0.05; Supplemental Table 3). Proteins with significantly increased and reduced abundance were identified in patients with active TB infection (Figure 3A). In addition to the identification of proteins known to be regulated during the active TB immunopathology, such as CRP, serum amyloid A (SAA), S100A8, retinol binding protein 4 (RBP4), MMP14, and diverse apolipoproteins, novel proteins were found, such as disks large homolog 4 (DLG4), pulmonary surfactant-associated protein B (SFTPB), complement factor H related 5 (CFHR5), and secreted phosphoprotein 2 (SPP2).

**Figure 4:**
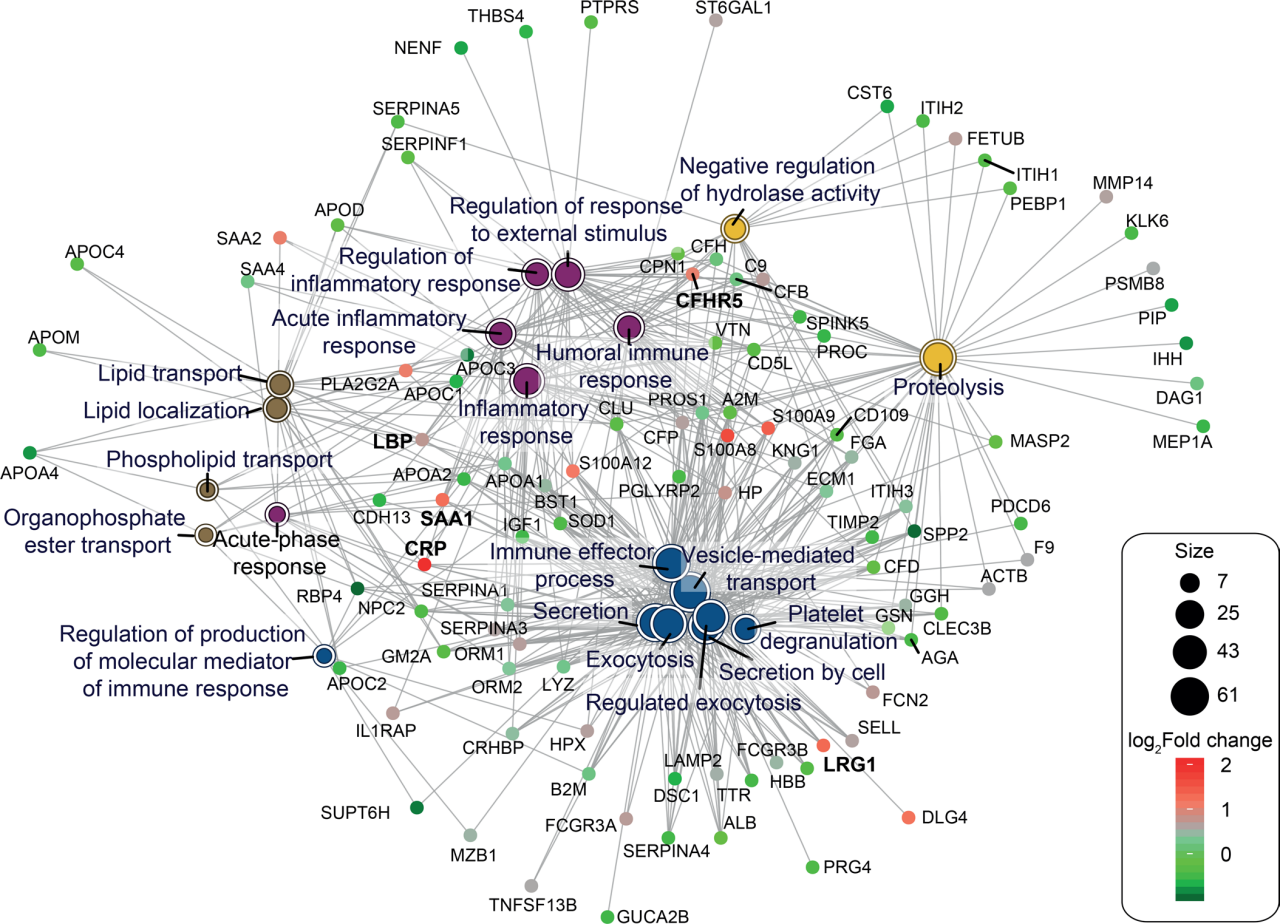
Physiological changes in pulmonary TB are reflected in the plasma proteome. Functional enrichment analysis of the biological processes was performed on the 189 proteins strongly associated with the TB status and identified by WGCNA. Gene concept network (cnet plot) depicts the linkages of proteins and the top 20 biological process terms enriched in the turquoise module. Upregulated and downregulated proteins were included. Green-to-red coding next to the network indicates the log_2_ fold change. Proteins in bold were selected for validation. LBP, lipopolysaccharide binding protein; LRG1, leucine rich alpha-2-glycoprotein 1; SAA1, serum amyloid A1.

Further data mining of the output from segment 4 was performed to interpret biologically relevant patterns in pulmonary TB. Weighted gene coexpression network analysis (WGCNA) (41) was used to explore relationships between clusters of highly correlated proteins (color modules) and specific sample traits. Technical and biological variables of batch, smoking history, and ethnicity were evaluated as possible confounders in the data using hierarchical clustering. The resulting dendrogram demonstrated that disease status was the primary determinant of sample clustering (Supplemental Figure 5A). To select highly interconnected proteins exhibiting the strongest correlation with the disease status, detection of modules was performed (Supplemental Figure 5B). The dendrogram of the topological overlap matrix representing clusters of highly interconnected proteins with assigned color modules and association to particular traits demonstrated that the protein module turquoise was strongly associated with disease status (Figure 3B, Z** score = –0.87; *P* = 2 × 10^–7^). A total of 189 proteins were contained in the turquoise module (Supplemental Table 4), of which 129 (65.8%) were common to the differentially expressed proteins defined with limma (7 protein unique to limma and 60 unique to WGCNA). GO enrichment was performed using the package clusterProfiler (42) on the turquoise module and demonstrated that proteins profiled were mainly associated with a variety of intracellular and secretory vesicles, extracellular matrix, blood microparticles, and lipoprotein particles (Figure 3C). Analysis revealed 4 main hubs for the top 20 biological processes: inflammatory/acute-phase response, exocytosis/vesicle-mediated transport, lipid transport, and proteolysis (Figure 4).

**Figure 5:**
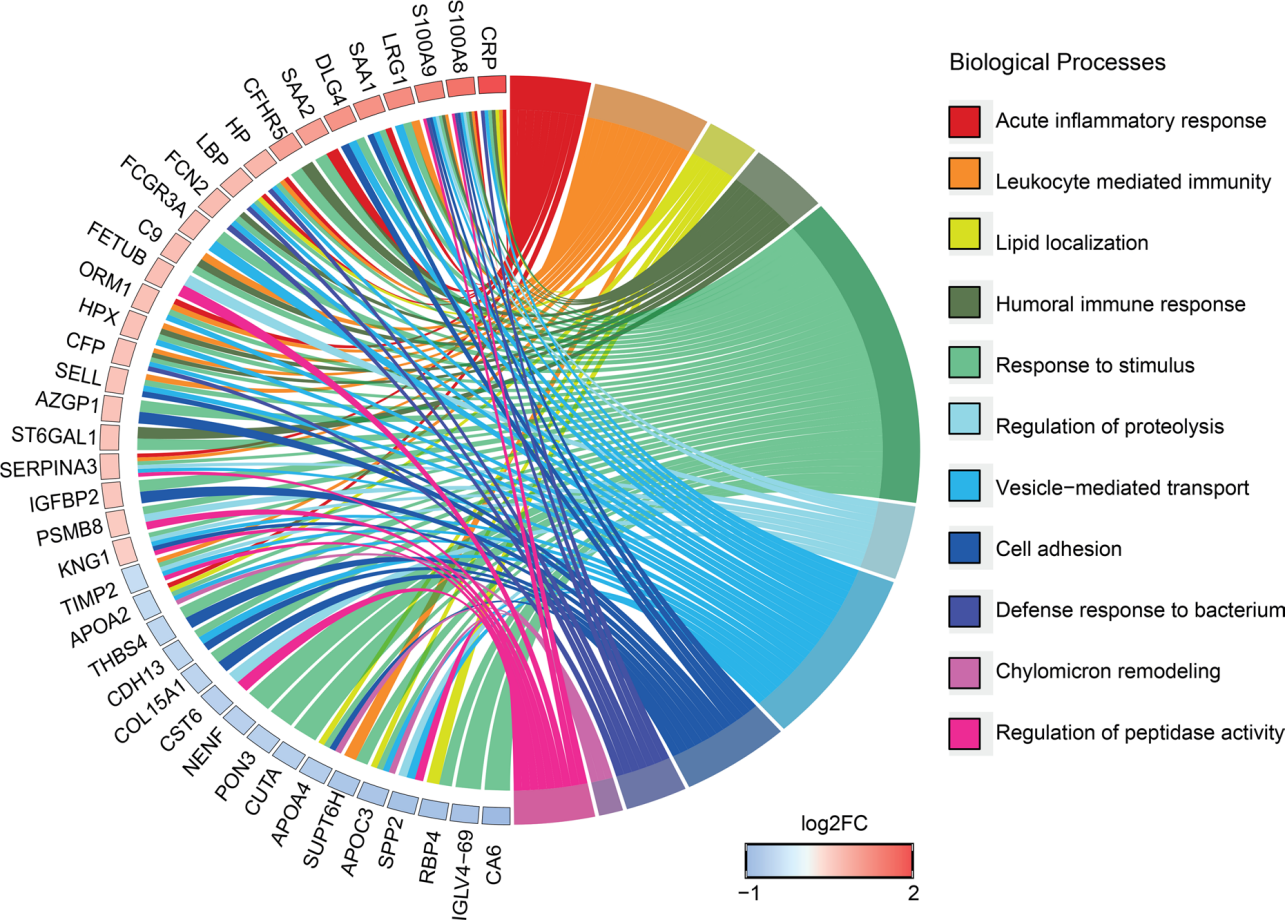
Top candidate biomarkers for active TB link to multiple biological processes. Chord plot for plasma proteins strongly correlated to active TB and identified by combining outputs from WGCNA and limma. This plot links these proteins via ribbons to their associated biological processes. Blue-to-red coding next to the proteins indicates the log_2_ fold change. GO enrichment for biological process was performed in g:Profiler, and only significant terms (FDR *q* ≤ 0.05) are shown. Plot generated with the R package GOplots.

To generate the most robust list of candidates for validation, we identified proteins in common between the module turquoise derived from WGCNA and significant by empirical Bayes moderated t-statistics in limma, thereby combining coexpression analytical approaches and t-statistics. Combining the approaches, we identified 26 common proteins with increased and 20 proteins with reduced abundance, with a high predicted significance (full list, Supplemental Table 5; log_2_ fold change ≥ |0.5|; WGCNA: *Z* score ≥ |0.65| and *P* ≤ 0.05; limma: *q* ≤ 0.05). This highly stringent approach is likely to omit numerous other differentially regulated proteins but maximizes the chance of subsequent validation for diagnostic use. Proteins in this list are associated with a wide range of biological processes, including acute inflammatory response, defense response to bacterium, lipid localization, cell adhesion, and regulation of peptidase activity (Figure 5).

### Host plasma proteins exhibit increased abundance in TB and other respiratory diseases.

Circulating levels of 5 proteins among the top 15 proteins with increased expression levels (Supplemental Table 5) were subjected to independent verification with ELISA or Luminex array. CRP and SAA1 were included in the verification panel because these are considered established major acute-phase effectors and are expected to increase in individuals with pulmonary TB. LBP and LRG1 have been described in other proteomic TB profiles (11, 43, 44); therefore, the expression of these proteins in specific cohorts may add valuable information for the design of a multimarker panel. Newly identified proteins from our analysis, such as CFHR5, were additionally selected for verification. Proteins closely biologically associated with the selected proteins were excluded for further verification, such as SAA2, since independency is recognized to benefit performance of multimarker panels. In addition to these selected candidates, the 7 most consistently divergently regulated proteins, analyzed by fold change, derived from the profile of HP-SEC segments 1 to 3, protein fantom (RPGRIP1L), fibrinogen-like protein 1 (FGL1), cartilage oligomeric matrix protein (COMP), small conductance calcium-activated potassium channel protein 2 (KCNN2), tumor necrosis factor ligand superfamily member 11 (TNFSF11), E3 ubiquitin-protein ligase listerin (LTN), and interleukin enhancer binding factor 2 (ILF2), were included to compare verification efficiency between the smaller and larger discovery groups.

**Figure 6:**
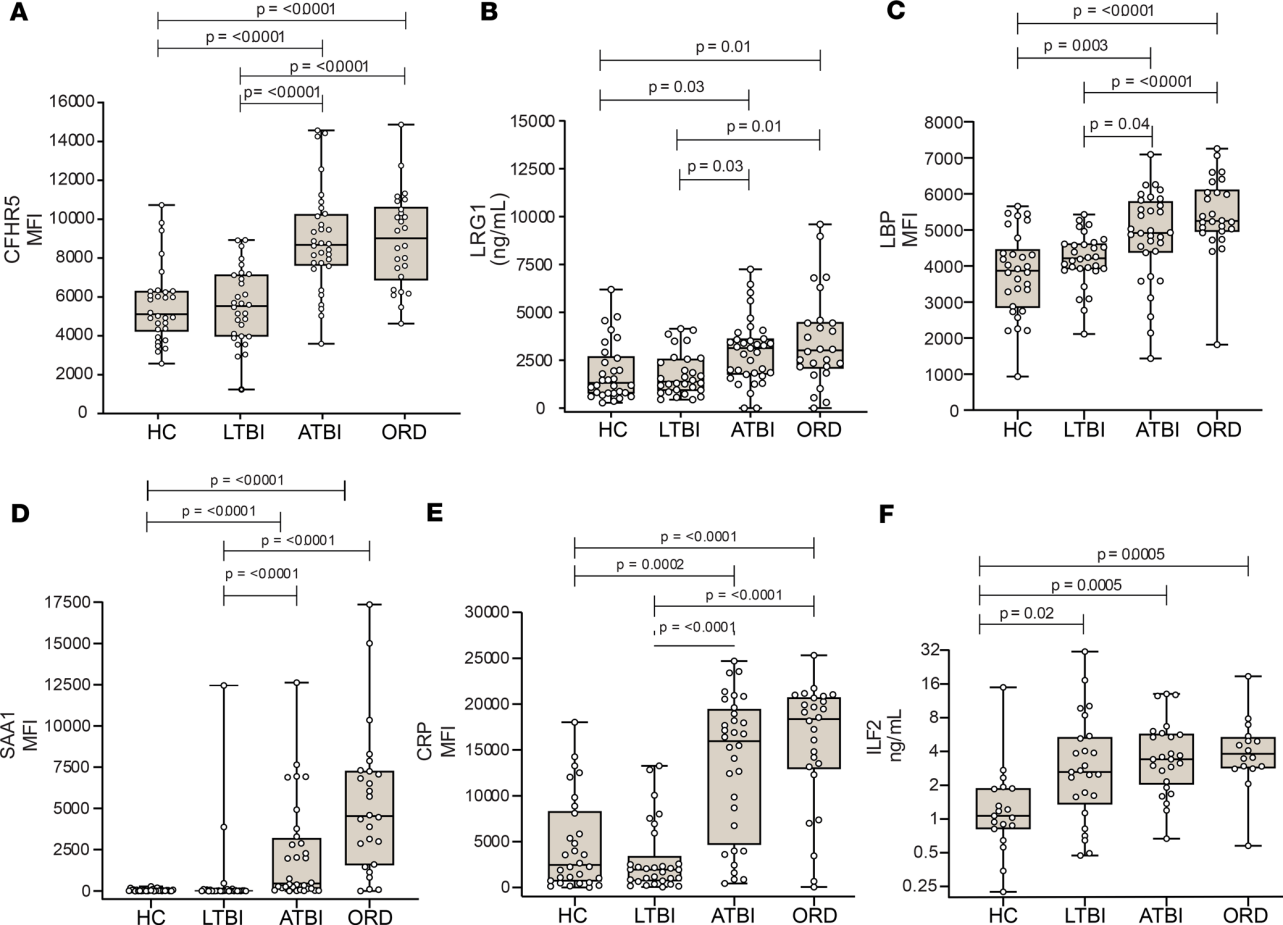
Novel TB biomarkers are validated in an independent UK cohort of mixed ethnicity. Two novel TB biomarkers were significantly upregulated in TB infection measured by Luminex or ELISA in serum from an independent UK-based cohort. (**A**) CFHR5 is increased in TB and also significantly increased in other respiratory diseases (ORDs). ATBI, active TB infection. Four known TB potential markers were measured and were significantly elevated in TB: (**B**) LRG1, (**C**) LBP, (**D**) SAA1, and (**E**) CRP. (**F**) ILF2, a novel analyte from segment 3, was elevated in TB and ORDs. Box displays 25% and 75% percentiles with line showing median and whiskers displaying minimum to maximum values. Differences were considered significant when *P* < 0.05 and calculated from Kruskal-Wallis test and Dunn’s multiple-comparisons test. HC, healthy controls (*n* = 30); LTBI, latent TB infection (*n* = 30); PTBI, pulmonary TB infection (*n* = 32); ORD, other respiratory diseases (*n* = 26).

First, we studied a UK-recruited independent cohort of mixed ethnicity from the Multifunctional Integrated Microsystem for rapid point-of-care TB IdentifiCation (MIMIC) study, for verification of selected candidates. CFHR5, LRG1, LBP, SAA1, and CRP showed significantly increased levels of expression in patients with active TB when compared with healthy controls or latently infected individuals (Figure 6, A–E). Evaluation of the markers selected from the initial discovery experiment on 7 samples showed that RPGRIP1L, FGL1, COMP, KCNN2, and TNFSF11 failed verification (Supplemental Figure 6). LTN (Supplemental Figure 7, P** = 0.04) abundance was significantly higher in patients with TB. Additionally, ILF2, identified from segment 3 analysis, showed elevated abundance in patients with latent TB and active TB compared with healthy donors (Figure 6F, P** = 0.0005). Consequently, 2 out of 7 proteins were successfully verified from the smaller discovery group, whereas all were verified from the larger discovery group. In addition to the proteins being elevated in TB, patients with ORDs also exhibited elevated abundance in all verified markers (Figure 6, A–F).

**Figure 7:**
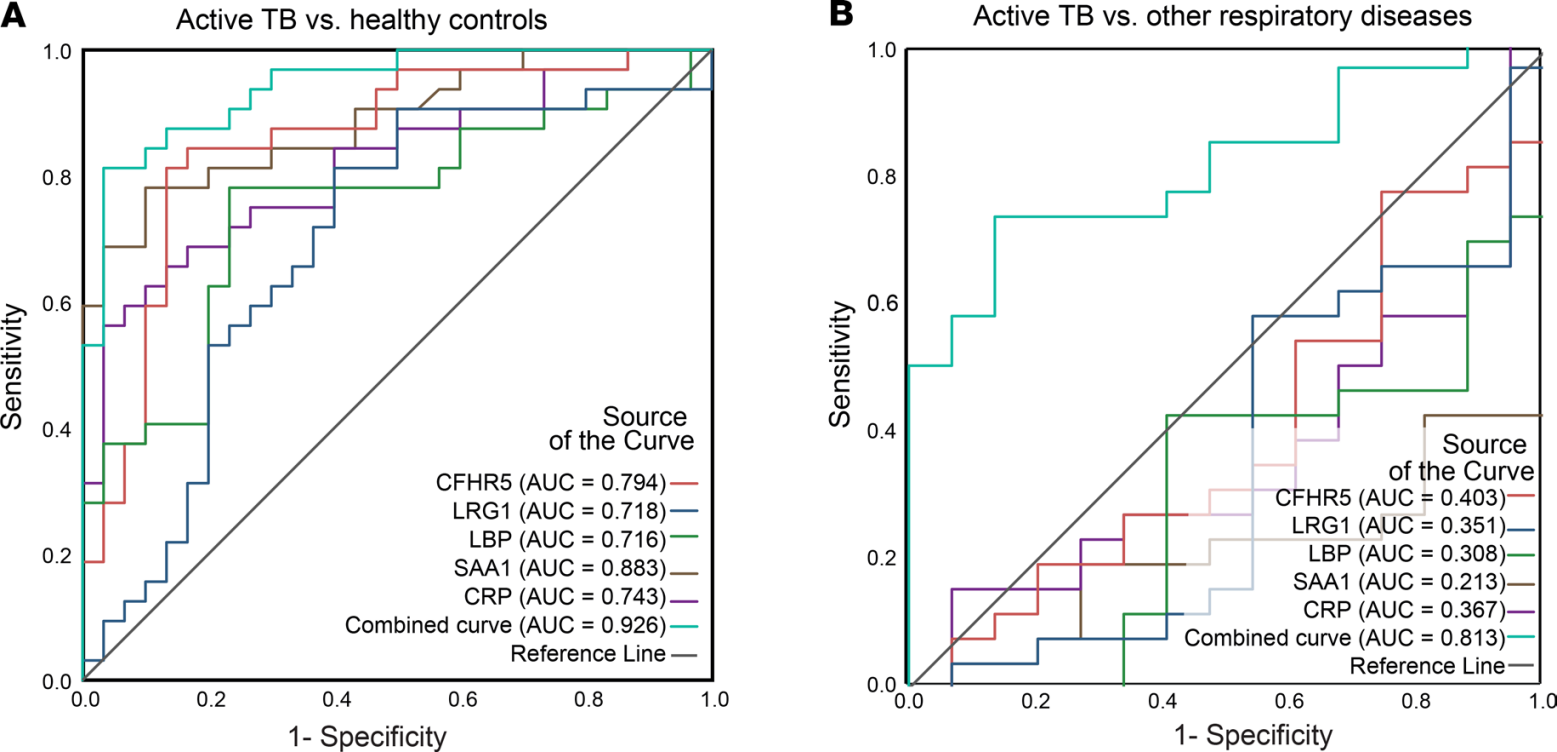
Combination of 5 protein markers discriminates patients with TB in a UK-based cohort. ROC curves were generated using SPSS v.25, for individual proteins (CFHR5, LBP, SAA, CRP, and ILF2) and after binary logistic regression for combined analytes. AUC was estimated under nonparametric assumption. TB was set as the positive test outcome and the test direction such that larger test result indicates a more positive test. ROC curve for TB infection versus HCs shows good discrimination, with the multiplex panel most discriminatory (**A**), while the ROC curve for TB infection versus ORDs shows individual analytes are not differentiating, but a combined multiplex panel generates an AUC of 0.813 (**B**).

Diagnostic performance of individual and combined verified markers was evaluated using receiver operator characteristic (ROC) curves. ROC curves were generated based on 2 different comparisons: circulating level of markers in patients with active TB versus HCs (Figure 7A) and patients with active TB versus ORDs (Figure 7B). In both cases, the best performance was achieved by combining the 5 markers (CFHR5, LRG1, LBP, SAA1, and CRP). The AUC was 0.93 (95% confidence interval: 0.89–1.00, *P* ≤ 0.001) for TB versus HCs and 0.81 (95% confidence interval: 0.68–0.94, *P* = 0.001) for TB versus ORDs, thus demonstrating that only the combination of markers allowed the discrimination of active TB from HCs and ORDs. Although ILF2 abundance was significantly upregulated in the active TB and ORD patients from this cohort (Figure 6F), it did not contribute toward a better diagnostic performance of the panel.

**Figure 8:**
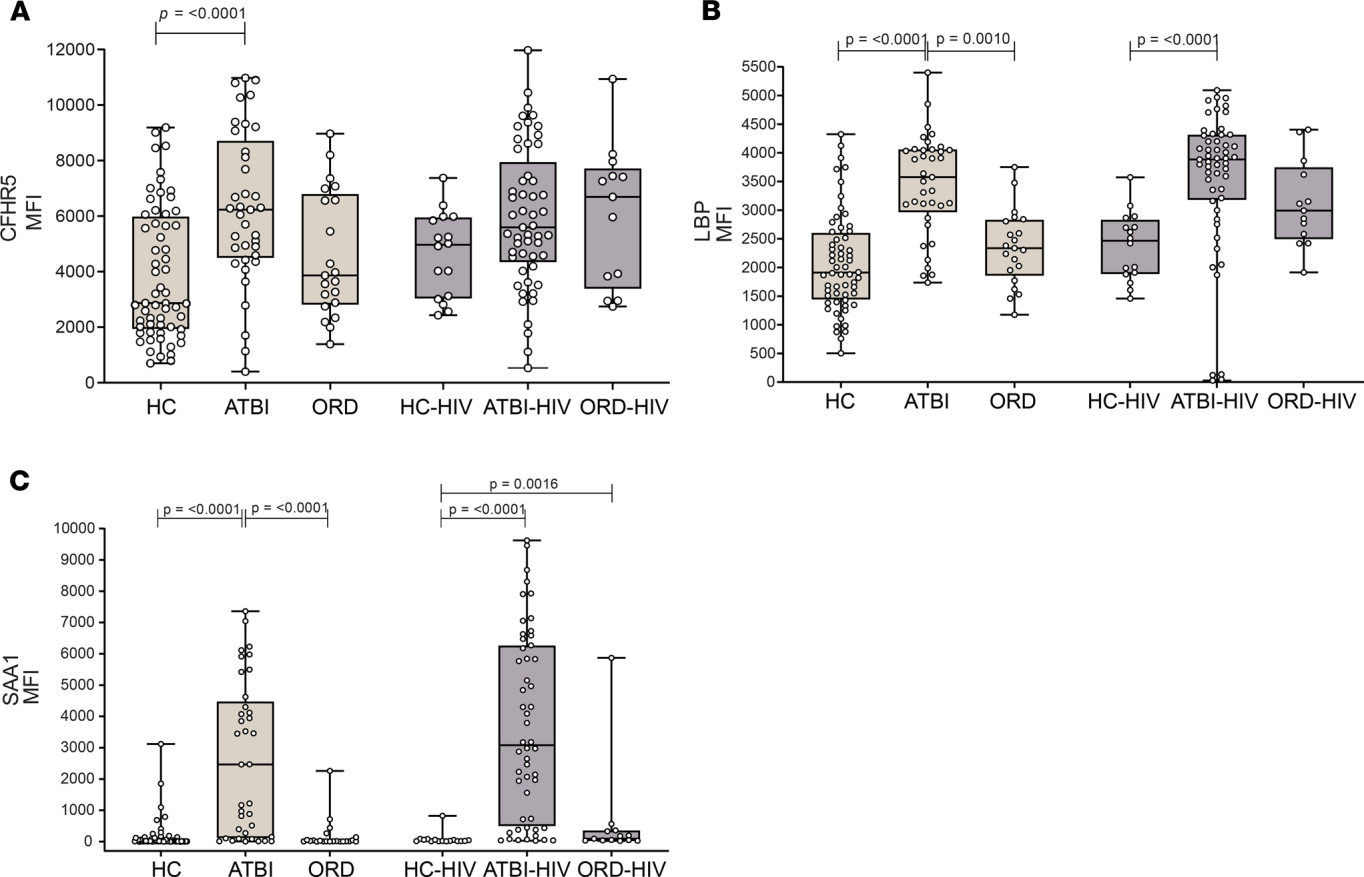
CFHR5 is validated as a new diagnostic marker of TB in HIV coinfection, and multiplex analysis performs well against other respiratory conditions. (**A**) CFHR5 was significantly upregulated during ATBI in a previously reported South African cohort, in both HIV-uninfected and HIV-infected individuals. Three other potential TB markers were also elevated: (**B**) LBP, (**C**) SAA1, and CRP (previously reported). Box displays 25% and 75% percentiles with line showing median and whiskers displaying minimum to maximum values. Differences were considered significant when *P* < 0.05 and calculated from Kruskal-Wallis test and Dunn’s multiple-comparisons test. HC-HIV (*n* = 16), ATBI-HIV (*n* = 53), and ORD-HIV (*n* = 13). HC, healthy controls (*n* = 60); PTBI, pulmonary TB infection (*n* = 39); ORD, other respiratory diseases (*n* = 22); HIV, HIV coinfection.

We then further verified the biomarkers in a South African cohort, which included HIV-uninfected and HIV-infected patients with active TB and ORDs. Again, the novel diagnostic marker CFHR5 exhibited significantly increased abundance in HIV-uninfected patients. In HIV-coinfected patients, CFHR5 was elevated compared with HCs, but not significantly different from healthy HIV-infected individuals, although this group had limited numbers (Figure 8A). CFHR5 showed no significantly increased abundance in ORDs, irrespective of HIV status. Again, the interpretation may be due to limited sample numbers reducing statistical power. LBP and SAA1 both showed increased abundance in the active TB group regardless of HIV status. This trend was observed relative to the ORD group HIV un- and coinfected (Figure 8, B and C). CRP showed increased abundance in TB compared with HC and ORD groups, irrespective of HIV status (cohort data previously published, ref. 7). In this cohort, ILF2 and LRG1 could not be measured because of sample exhaustion and were thus excluded from the panel. A summary of the analytes tested in each cohort and verification results is presented as Supplemental Table 6.

**Figure 9:**
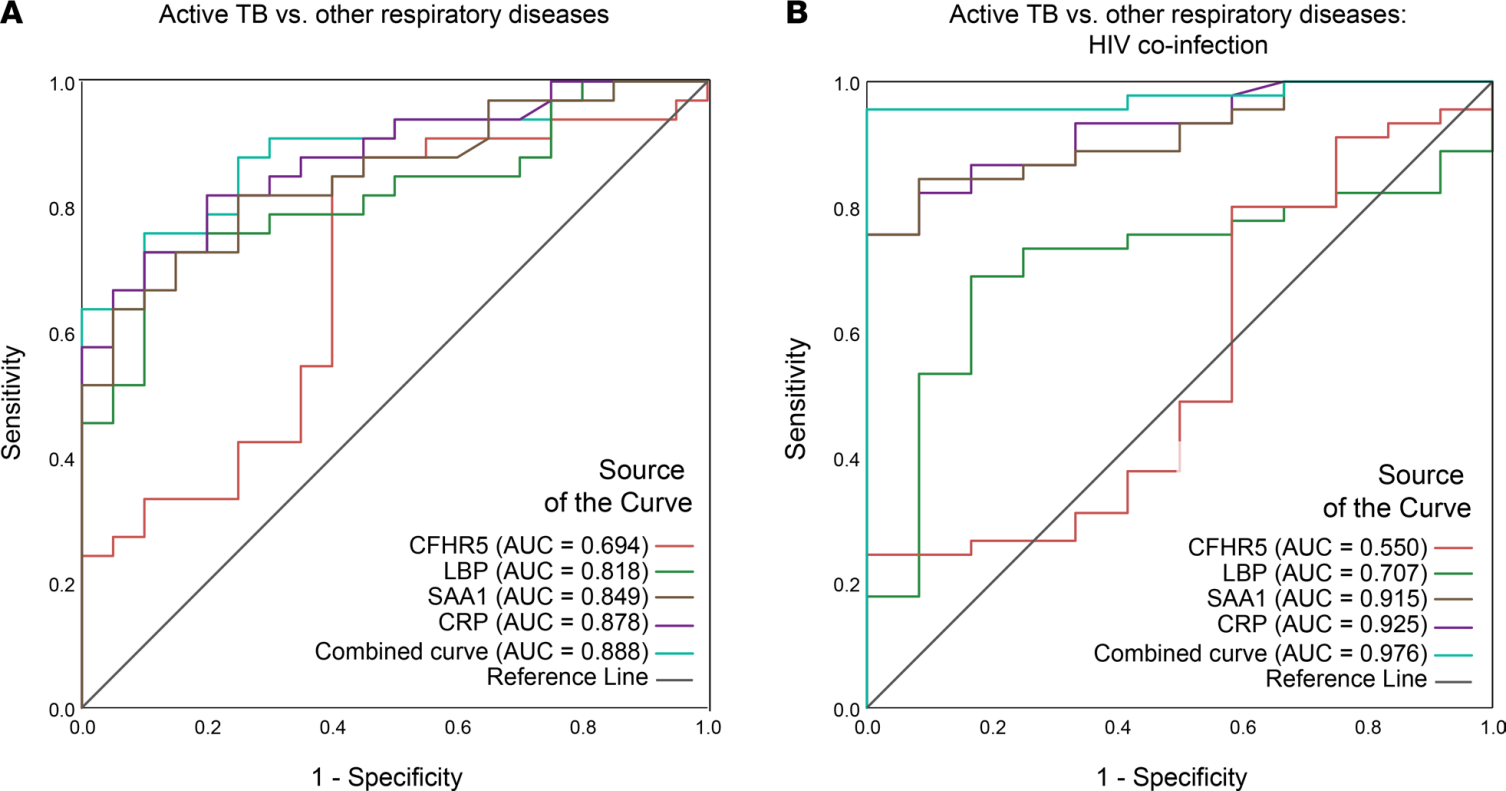
Combination of 4 protein markers discriminates TB patients with HIV coinfection. ROC curves were generated using SPSS v.25, for individual proteins (CFHR5, LBP, SAA1, and CRP) and after binary logistic regression for combined analytes. ROC curve for TB infection versus ORDs in HIV-uninfected individuals shows optimal performance from the combined host panel, with AUC of 0.888 (**A**). Analysis of TB infection versus ORDs in HIV-coinfected individuals produced an AUC of 0.976 from the combined panel (**B**).

ROC curves generated by comparing circulating levels of CFHR5, LBP, SAA1, and CRP in TB patients versus ORDs in the HIV-uninfected group showed that the best performance was achieved by combining markers (Figure 9A, AUC 0.89 [95% confidence interval: 0.80–0.98, *P* ≤ 0.001]). Similarly, in the context of HIV-associated TB, the combination panel performed best and provided a surprisingly high discrimination between active TB and ORDs (Figure 9B, AUC 0.98 [95% confidence interval: 0.94–1.00, *P* ≤ 0.001]). By contrast, the combination of markers did not improve the diagnostic performance when the active TB group was analyzed against the HCs relative to analysis of CRP alone (Supplemental Figure 8). Finally, we evaluated whether our 4-protein panel correlated to sputum mycobacterial load in the South African cohort. Mean *Z* scores were calculated from CFHR5, LBP, SAA1, and CRP levels in patients with TB (HIV negative) and compared with the bacterial burden in sputum. A significant positive correlation was observed (Spearman’s coefficient *r* = 0.37, *P* = 0.03).

## Discussion

We applied a unique nondepletion-based quantitative proteomics method (q3D LC-MS) to generate the most comprehensive TB plasma proteome to date. Statistical power was increased by studying 1 HP-SEC segment in additional patients, and combined WGCNA and limma analysis approaches identified numerous novel host biomarkers with high confidence. We verified a subset of biomarkers in 2 separate cohorts, with a high success rate. Diagnostic accuracy for TB was maximized by use of a multimarker panel. These markers are frequently also increased in other respiratory conditions, and therefore host biomarkers are likely to be of greatest use in a rule-out panel.

Translation of novel biomarkers for clinical utility is challenging, involving a stepwise process where most candidates fail to reach the bedside. Verification of new candidates typically relies on antibody-based assays, requiring change of platform from mass spectrometry to immunoassays before field-testing, and this is frequently a point of failure. We completed this transition for 3 new analytes, thereby supporting the robustness of the approach. Validation will require quantification of the additional 15 entirely new biomarkers in the top candidates (Figure 5, Supplemental Table 5) identified by the combined WGCNA and limma approaches and interlaboratory collaboration across large cohorts from multicenter biobanks, including analysis of how biomarkers relate to disease severity and change over time.

Plasma is a complex matrix to analyze, and high-abundance protein depletion is the most common strategy to address this complexity (5, 27–29, 45, 46). However, depletion may inadvertently coremove important analytes noncovalently bound to high-abundance proteins (26). In this study, sample preparation was principally based on the use of orthogonal chromatographic hyperfractionation instead of depletion. Such a strategy entailed the dissolution of 120 μL neat plasma with 7 M guanidine/10% methanol that stabilized the protein content and was subjected to HP-SEC separation as part of the hyperfractionation pipeline. The use of multidimensional liquid chromatographic approaches as part of the isobaric quantitative proteomics pipeline has gained increasing prominence in translational research studies (47). Such approaches compensate for the complexity of biological specimens in capturing and analyzing very low-abundance proteins of clinical significance. Furthermore, they are amenable to laboratory automation and scale-up, thus improving analysis throughput, accuracy, and precision (47, 48). In line with this, the collective attributes of the present study method facilitated the analysis of proteins encompassed in blood microparticles, such as exosomes and other lipid vesicles (27, 28), along with protease-derived cleavage proteins and soluble proteins. The efficacy of our approach was demonstrated by the profiling of over 5000 proteins from only 120 μL plasma per patient, compared with the identification of a maximum of 800 proteins in similar TB discovery studies from larger volumes of plasma (16, 20, 49). Most importantly, however, the deep proteome coverage achieved also coded for a wide spectrum of biological and disease-specific pathways and networks of physiological relevance to TB. Encompassed in these pathways and networks were many novel proteins of potential clinical significance.

Analysis of the entire proteome from HP-SEC segments 1 to 4 using 7 samples was underpowered for biomarker discovery, with only 2 out of 7 candidates subsequently being validated in a larger cohort. Therefore, detailed profiling was focused on the subproteome segment 4, which is primarily enriched for low–molecular weight proteins and protein degradation products, recapitulating multiple biological processes (28, 29, 50–52). In-depth profiling of this segment from 10 HCs and 11 pulmonary TB patients provided much greater statistical power, consistent with mathematical estimations (38). The high-dimensional data produced from isobaric labeling-based relative quantification (iTRAQ or tandem mass tag) poses bioinformatic processing challenges (34). Small sample sizes, incomplete data sets, and batch effects across experiments create difficulties in the effective detection of protein abundance changes (35). Batch effects are particularly relevant to multiplexing of iTRAQ experiments. In our study, ComBat correction performed better than the most common strategy of normalizing to a common reference sample (Supplemental Figure 4). Complementary analysis using limma and WGCNA on the adjusted data resulted in a powerful approach producing a set of robust markers for verification (Supplemental Table 5), with 3 out of 3 tested proteins successfully converting to an immunoassay platform, compared with 2 out of 7 from the smaller sample set (Set A profile). Thus, this methodology led to the identification and independent verification of known and novel candidate biomarkers of TB infection.

WGCNA identified 1 coexpression module as strongly associated with the group TB (turquoise module, *P* = 2 × 10^–7^), containing 189 proteins. Ninety-five percent of the differentially expressed proteins identified with limma were common to this module, showing excellent concordance between analytical strategies. Notably, over 60% of the coexpressed proteins showed decreased abundance in the active TB group, suggesting that studying these proteins may provide additional insight into disease processes in TB, and analysis should not purely focus on proteins of increased abundance. GO enrichment of module turquoise revealed regulation of biological processes associated with responses to external stimulus (*q* = 2 × 10^–3^) encompassing acute-phase/inflammatory (*q* = 5.2 × 10^–6^) and humoral responses (*q* = 9.2 × 10^–5^). Within this module, CRP, LBP, SAA1, SAA2, S100A8, S100A9, SERPINA3, and HP are involved in the activation of the acute-phase and inflammatory response, which are well described in TB (20, 53, 54). This concordance supports the overall validity of our methodology.

Connected to the acute-phase hubs, proteolysis (*q* = 1.1 × 10^–6^) and lipid transport and localization (*q* = 1.4 × 10^–5^) were significantly enriched. Proteolysis is consistent with the extensive pulmonary destruction that occurs in human TB (55). Among the proteins with increased abundance in this hub, ECM1 was previously reported as elevated in saliva of patients with TB (56), MMP14 is expressed in TB granulomas (57), and PSMB8 may be part of the regulatory cascade of the blood transcriptome of patients with TB (58). Among the proteins found with decreased abundance, TIMP2 is an inhibitor of matrix metalloproteinases, and so reduced levels may increase matrix degradation (55). Lipid metabolism was another major signal expressed, and the role of lipids and cholesterol in TB immunopathology remains poorly characterized. Cholesterol uptake and catabolism are central for maintenance of the pathogen in the host and contribute to pathogenesis and virulence (59). However, the low circulating lipid profiles in patients with pulmonary TB may be a consequence of the disease or may have wider biological implications. Apolipoproteins are associated with lipid transport and form lipoprotein particles such as HDL, LDL, and VLDL. Serum HDL-C concentrations negatively correlate with the radiological extent of disease and smear positivity in pulmonary TB (60). Decreased circulating concentrations of apolipoproteins are consistently reported in different serum/plasma proteomic profiles for pulmonary TB (11–13), in agreement with our findings. Further data mining of these biological processes may identify host-directed therapy targets.

To verify newly identified biomarkers, well-characterized TB cohorts with complementary profiles and from geographically diverse populations are required (4). We studied 2 different cohorts for verification, 1 recruited in the United Kingdom and 1 in South Africa. From the subset of proteins analyzed by ELISA or Luminex, 7 proteins were successfully validated. LBP, CFHR5, CRP, and SAA were consistently increased in TB cases in both cohorts. Statistically significant differences were observed despite the wide interindividual variation in biomarker concentrations, which is expected from clinical TB, which has a wide spectrum of disease severity. ILF2 was only verified in the MIMIC cohort because of sample exhaustion, while LTN and LRG were only evaluated in the South African cohort. CFHR5, ILF2, and LTN are novel protein candidate biomarkers for TB identified by the discovery phase and all were successfully verified. Consistent with our findings, a recent report identified ILF2 as a potential biomarker in pediatric TB by bioinformatic mining of gene expression data sets (61).

Evaluation of the performance of a subset of markers indicated that combination rather than individual markers provided a better diagnostic ability. In the UK-based cohort, ROC analysis demonstrated that the multimarker panel comprising CFHR5, LRG1, CRP, LBP, and SAA1 performed well in ROC analysis against HCs (AUC = 0.93). However, the discriminatory power was reduced but still significant when compared against ORDs (AUC = 0.81). Clinically, differentiation against other respiratory conditions is the key comparator for TB diagnosis. Host biomarkers are often limited by lack of specificity, and our findings reinforce the importance of choosing correct control groups for verification analysis (18). In the South African cohort including patients with and without HIV infection, the multimarker panel comprising LBP, CFHR5, CRP, and SAA yielded its best performance when patients with TB were compared with ORDs (AUC = 0.98). This is an important finding from a clinical perspective, as diagnosing TB in HIV-infected patients is generally more challenging than in nonimmunocompromised individuals (2). Furthermore, performance of our panel in both cohorts (United Kingdom and South Africa) comparing ATBI to ORD groups was similar to a different recently validated host response signature (IL-6, IL-8, IL-18, and VEGF, AUC = 0.80) (62). This suggests our preliminary signature can be further refined by testing of remaining highly significant candidates that have not yet been studied. The primary difference between the groups is that the UK cohort were hospitalized patients, whereas the South African cohort were outpatients, and therefore the better performance in South Africa may reflect the fact the patients were less unwell. For utility of a point-of-care test, outpatients with respiratory symptoms will be the primary target group.

Significant efforts have been directed toward defining an optimal plasma protein biosignature for active TB, and recently, extensive testing of candidate proteins identified by predefined discovery panels, such as those measured with Luminex, has shown that multicomponent or multifactorial signatures could give a greater performance than immunological markers despite the heterogeneity of clinical presentation (62, 63). Inclusion of novel markers that represent the biological diversity of the host response to the *M. tuberculosis* infection in diagnostic panels may be crucial to achieve the analytical performance required to translate to effective point-of-care devices. From our top list of 46 proteins identified by both limma and WGCNA from the discovery phase (Supplemental Table 5), 21 proteins are entirely novel candidates and involved in a wide range of biological processes. Consequently, verification and integration with known markers may improve the performance of the existing signatures. This list recapitulated several potential diagnostic biomarkers identified in a range of reported plasma proteomic TB signatures (11, 13, 14, 20, 44, 64), including 1 signature for TB progression (17), 1 for cured pulmonary tuberculosis (21), and 1 for multidrug-resistant TB (65), demonstrating the ability of our proteomic and bioinformatic approach to detect proteins associated with the disease status, independent of differences in discovery platforms or patient cohorts. However, further verification of all the candidates that we identify here is required to refine the current panel.

Translation of such markers to point-of-care tests with adequate performance will require the development of multiplex lateral flow assays, and such platforms are currently emerging (66, 67) yet will require careful development. Any assay used as a rule-out test would need population-based studies to confirm the specificity against standard current clinical practice and emerging blood protein-based signatures. Due to the overlap between TB and other respiratory conditions, the host biomarkers identified are potentially best utilized as a rule-out triage test before performing more specific and expensive rule-in tests (68). In the future, analysis of other proteins that are differentially abundant will become increasingly achievable, given the continuous advancements of LC-MS methods in terms of throughput and analytical confidence. When combined with machine learning approaches, LC-MS–based assays may transform specificity and sensitivity in the diagnosis of TB.

In summary, we developed a nondepletion-based proteomic methodology to deeply profile plasma and identify novel biomarkers. We present a unique statistical and bioinformatic pipeline for discovery and selection of candidates for verification that uses both statistical significance and correlation of expression patterns to clinical traits. We report numerous novel analytes, with potential to be translated for clinical utility. We have verified a subset of biomarkers from segment 4 by independent antibody-based assays to generate a preliminary diagnostic panel, and similar interrogation of segments 1 to 3 is likely to generate further novel biomarkers. Taken together, developing these host biomarkers into a multiplex lateral flow assay has potential for a near-patient TB rule-out test that fulfills the WHO product characteristics. Such an assay could be a powerful tool to address the global TB pandemic.

## Methods

### Study participants

This study included participants from 3 different cohorts. The participants from the South African cohort were recruited at Ubuntu HIV/TB clinic in Cape Town from June 2012 to February 2014 and were of Black African ethnicity. Written informed consent was obtained, HIV testing was offered, and chest radiographs were performed as per routine practice. The diagnosis of active TB was based on sputum smear or culture positivity, Gene Xpert results (where available), and chest x-ray examination. For the control group, all sputum samples were smear and culture negative for acid-fast bacilli. Plasma samples from this cohort were retrospectively selected from a cohort collected and previously described (7). Participants from this cross-sectional study were categorized into 6 groups: (i) HIV-uninfected patients without ATBI (HIV^–^ ATBI^–^), (ii) HIV-uninfected patients with ATBI (HIV^–^ ATBI^+^), (iii) HIV-uninfected patients without active TB but with symptoms attributable to other respiratory infectious disease (HIV^–^ ORD), (iv) HIV-infected without ATBI (HIV^+^ ATBI^–^), (v) HIV-infected with ATBI (HIV^+^ ATBI^+^), and (vi) HIV-uninfected patients without active TB but with symptoms attributable to ORD (HIV^+^ ORD). Microbiological confirmation of the infectious agent was not available for the HIV^–^/HIV^+^ ORD groups because of limitations in local diagnostic capability. A randomly selected subset of 11 plasma samples from male participants belonging to the groups HIV^–^ ATBI^–^ and HIV^–^ ATBI^+^ was used for discovery (Supplemental Table 1). A larger set of 203 samples from all 6 groups and including those used for discovery constituted the South African verification cohort, and the demographic description of this group has been previously reported with a CONSORT diagram (7).

Participants from the Peruvian discovery cohort were prospectively recruited at clinics in Lima, Peru, to match demographic features such as sex, age, and BMI of participants from the South African cohort. Recruitment was conducted during 2015. The diagnosis of active TB was based on a TB symptom questionnaire, sputum smear positivity, culture positivity using microscopic observation drug susceptibility culture, and chest x-ray. Healthy control individuals were Quantiferon negative. In total, 10 samples from this cohort were selected for the discovery stage of this study (Supplemental Table 1).

A second independent cohort was included for verification of proteomic candidates comprising a subset of 118 participants from the MIMIC cross-sectional study conducted in the United Kingdom. Recruitment was performed from June 2014 to February 2017. All the participants were HIV uninfected, and 4 categories were defined for this cohort: (i) HCs, (ii) LTBI, (iii) ATBI, and (iv) ORDs. HCs were asymptomatic individuals without a history of previous active TB or TB contact and no evidence of TB infection on routine screening tests (negative IFN-γ release assay and/or tuberculin skin test result). Participants with LTBI were defined based on a positive IFN-γ release assay and/or tuberculin skin test result, without evidence of active disease after clinical evaluation. All active pulmonary TB cases were individuals with symptomatic respiratory infection that were microbiologically confirmed to have TB based on any of the following criteria: sputum smear positive, sputum culture positive for *M. tuberculosis*, or PCR test positive for *M. tuberculosis*. The control group ORDs were symptomatic individuals with microbiologically confirmed respiratory tract infection caused by a pathogen (viral or bacterial) other than *M. tuberculosis*, without a history of previous active TB (Supplemental Table 7). The microbiological composition of this group was 31% influenza A/B, 15% *Streptococcus pneumonia*, 8% respiratory syncytial virus, 8% *Staphylococcus aureus*, 4% *Mycoplasma pneumonia*, 4% human metapneumovirus, 4% H1N1 influenza A, 4% methicillin-resistant *Staphylococcus aureus*, and 22% unidentified organism.

### Plasma processing

Venous blood was collected in sodium citrate vacutainer tubes and plasma prepared according to standard operating procedures at the site of recruitment and stored at –80°C. Aliquots of 120 μL of plasma were liquid fixed with 380 μL of 7 M guanidine hydrochloride and 10% methanol and stored at –20°C until SEC fractionation was performed for the discovery stage. Aliquots of 20 μL of the individual samples available for discovery including control and active TB groups was combined to generate a master pool aimed to control batch effects across different MS experiments. All the plasma samples included in the verification stage were divided into 100 μL aliquots to reduce freeze-thaw cycles when received and stored at –80°C until analysis.

### Multidimensional plasma proteomic analysis

#### High-performance size exclusion chromatography.

A general overview of the plasma proteomic method is presented in Supplemental Figure 1A. Plasma samples used for discovery, including 4 aliquots of the master pool, were individually subjected to HP-SEC prefractionation under optimized conditions of the method reported previously (28). Five columns were serially connected: 2 Shodex KW-804 columns, 8.0 mm internal diameter (I.D.) × 300 mm; 1 Shodex KW-802.5 column, 8 mm I.D. × 300 mm; and 2 Shodex KW-804 columns, operated at 45°C and 1.5 mL/min under isocratic elution with 6 M guanidine hydrochloride and 10% methanol. Four protein HP-SEC segments were collected in a peak-dependent fashion detected at 280 nm and then stored at –20°C until further analysis. HP-SEC separations are presented in Supplemental Figure 2, A–E. The BEH450 SEC Protein Standard Mix (Waters) and an aliquot of 1 control plasma sample were run for day-to-day quality control of the separation variation (Supplemental Figure 2F). Variation of retention times was within 2SD for all samples excepting 1 (Supplemental Figure 2G). Protein segments were dialysis purified using 3 kDa MWCO Slide-A-Lyzer cassettes according to manufacturer’s specifications (Thermo Fisher Scientific), with exchanges of 4 volumes of 4 L of ultrapure water every 12 hours in a cold room environment (4°C). The resulting dialysates were completely lyophilized using the Edwards Modulyo EF4-174 freeze dryer and Thermo Savant Micro Modulyo-115 benchtop freeze dryer. Protein extracts were stored at –80°C under argon atmosphere.

#### Trypsin digestion.

Total protein lyophilized extracts obtained from each HP-SEC segment were reconstituted with 0.5 M triethylamonium bicarbonate and 0.05% sodium dodecyl sulfate and sonicated on ice. Protein extracts were then centrifuged for 10 minutes at 16,000*g* and 4°C, and protein content in the supernatants was estimated using the NanoDrop ND-1000 spectrophotometer (Thermo Fisher Scientific) using the A280 program. Then, 120 μg of protein, volume adjusted, was reduced with 2 μL of 50 mM Tris-2-carboxymethyl phosphine and incubated for 1 hour at 60°C. Reduced samples were then alkylated using 1 μL of 200 mM methylmethane thiosulphonate and incubated 10 minutes at room temperature. Digestion was conducted to a ratio of 1:40 enzyme/substrate with trypsin MS grade (Pierce, Thermo Fisher Scientific) overnight for 16 hours at 37°C in the dark.

#### Stable isotope labeling.

iTRAQ 8-plex tags were equilibrated at room temperature, and isopropanol was added accordingly to ensure more than 60% organic phase during labeling. Each tag was added to the appropriate trypsinized sample; then the labeling reaction was conducted for 2 hours at room temperature. The reaction was stopped with 8 μL of 5% ammonium hydroxylamine. Samples were dried and stored at –20°C until chromatographic separation. The master pool was labeled using the tag 113, and the samples were allocated randomly to the remaining tags as presented in Supplemental Figure 2A.

#### Offline alkaline RP-HPLC peptide fractionation.

Offline peptide fractionation was based on high pH (0.08% v/v NH_4_OH) RP chromatography using the Kromasil C_4_ column (3.5 μm, 2.1 mm × 150 mm) and on the Shimadzu HPLC system previously described in the HP-SEC section. iTRAQ-labeled tryptic peptides were analytically reconstituted and pooled with 100 μL of mobile phase, then centrifuged at 16,000*g* at room temperature for 10 minutes. Supernatant was injected and separated at a flow rate 0.30 mL/min and 30°C. The fractions were collected in a peak-dependent fashion detected at 215 nm. Peptide fractions were dried at room temperature with a speedvac concentrator for 4–5 hours and stored at –20°C until LC-MS analysis. Highly hydrophilic and hydrophobic fractions from the extreme regions of the chromatographic traces were pooled and further cleaned using Gracepure SPE C18-AQ 100 mg/1 mL cartridges (Grace).

#### LC-MS analysis.

The LC-MS experiments were performed on the Dionex Ultimate 3000 UHPLC system coupled to the high-resolution nano-ESI-LTQ-Velos Pro Orbitrap-Elite mass spectrometer (Thermo Fisher Scientific). Higher energy collisional dissociation (HCD) and collision-induced dissociation (CID) fragmentation for each of the collected fractions was performed. For the analytical separation the AcclaimPepMap RSLC, 75 μm × 25 cm, nanoViper, C18, 2 μm particle column (Thermo Fisher Scientific) with trap cartridge retrofitted to a PicoTip emitter (FS360-20-10-D-20-C7) was used for multistep gradient elution. MS characterization of eluting peptides was conducted between 380 and 1500 *m/z*. The top 10 +2 and +3 precursor ions were further characterized by tandem MS (MS/MS). Full MS scans and MS/MS scans were acquired at a resolution of 30,000 full width at half maximum (FWHM) (complete plasma proteome) or 60,000 FWHM (detailed analysis segment 4) for profile mode and 15,000 FWHM for centroid mode, respectively, with the lock mass option enabled for the 445.120025 *m/z* ion (DMSO). Data were acquired using Xcalibur software (Thermo Fisher Scientific). Conditions for ionization, CID and HCD fragmentation, and ion detection were reported in a previous work (28).

#### MS data processing.

Target decoy searching of raw mass spectra data was conducted with the Proteome Discoverer 1.4 software (Thermo Fisher Scientific). SequestHT was used for the target decoy search for tryptic peptides, allowing 2 missed cleavages, 10 ppm mass tolerance, and minimum peptide length of 6 amino acids. A maximum of 2 variable (3 equal) modifications, oxidation (M), deamidation (N, Q), and phosphorylation (S, T, Y), were set as dynamic modifications, as static modifications were set: iTRAQ8plex (any N-terminal), Methylthio (C), and iTRAQ8plex (K). Fragment ion mass tolerance was set to 0.02 Da for the Fourier-transform–acquired HCD spectra and 0.5 Da for the ion trap–acquired spectra. FDR was estimated with Percolator (64 bit), and validation was based on *q* < 0.01 for high confidence or *q* < 0.05 for moderate confidence. All spectra were searched against a concatenated FASTA file including the reviewed UniProtKB SwissProt human proteome and the reference proteome (SwissProt and TrEMBL) for *M. tuberculosis* (strain ATCC 25618 / H37Rv), both retrieved on August 4, 2017. All peptide spectrum matches of reporter ions and iTRAQ ratios were exported to.txt at 1% FDR or 5% FDR peptide confidence and 50% coisolation exclusion threshold. Protein grouping was allowed and maximum parsimony principle was applied. Only unique peptides were considered for quantification downstream analysis. Raw precursor ion intensities from unique peptides were imported to R (version 3.3.1) and median adjusted. Median-normalized peptide intensities were log_2_ transformed, and values were averaged to obtain the mean relative expression for each protein. Only proteins with relative quantification reported in all the samples were included for statistical analysis. The MS proteomics data have been deposited to the ProteomeXchange Consortium via the PRoteomics IDEntification Database (69) partner repository with the data set identifier PXD020212.

### ELISA and Luminex assays

Proteins selected for verification from the proteomic discovery experiments were measured in 2 different cohorts using ELISA or Luminex assays. ELISA measurements comprised candidates for which there are commercially available kits, such as RPGRIP1L, FGL1, COMP, ILF2, KCNN2, LTN1, LRG1, and SFTPB (2B Scientific Ltd and Caltag Medsystems Ltd). One Luminex multiplex assay was custom-made for analysis of LBP, COMP, TNFSF11, and CFHR5 and 2 single-plexes for SAA1 and CRP (Protavio Ltd). The coefficient of variation for the ELISAs was no more than 12% and for the Luminex assays was no more than 15%. Assays were performed according to manufacturers’ directions.

### ROC curves and AUC analysis

Performance of the validated candidates was in the first instance assessed by calculating ROC curves for individual proteins and combined proteins in each verification cohort. The statistical package SPSS v.25 (IBM) was used for this purpose. ROC analysis was conducted by setting pulmonary TB as a positive test, and binary logistic regression probabilities were calculated when analysis of combined markers was performed. Coordinates of the curves were exported to estimate potential cutoff values.

### Statistics

Differentially expressed proteins were determined using linear modeling limma (70) followed by FDR correction for multiple-correction testing. WGCNA-based analysis was applied to the data sets resulting from the detailed profile of segment 4 to interpret biologically relevant patterns of protein expression in plasma of patients with pulmonary TB. The WGCNA R package was used to explore the correlation relationships between clusters of highly correlated proteins (color modules) and specific sample traits. The batch effect was corrected to increase the analysis power with ComBat (40). Networks of highly interconnected proteins were constructed using a soft-thresholding power of 0.9, and modules were identified using a minimum module size of 15. Module significance was calculated as a measurement of the correlation between biological traits, such as disease or group, ethnicity, and smoking status, and the protein expression profiles. Visualization tools available from this package were used to identify modules strongly correlated to biologically relevant covariates. Functional enrichment analysis was conducted using the option g:GOSt available in the tool g:Profiler (71). Only GO terms with an FDR-adjusted *P* value (cutoff 0.05) were considered. Significant GO terms were summarized by removing redundant terms using the tool REVIGO (72). We generated cnet plots using the R package clusterProfiler (73).

For ELISA and Luminex measurements, differences between groups were analyzed by Kruskal-Wallis test and using Dunn’s multiple-comparisons correction. Data were analyzed with Prism 8 (GraphPad). A *P* ≤ 0.05 was considered statistically significant. For the ROC analyses, the nonparametric method was used to estimate the standard error of the AUC, and the confidence interval was set at 95%.

### Study approval

All clinical studies were conducted according to Declaration of Helsinki principles. All participants gave written informed consent before inclusion in any of the clinical studies here included. The South African cohort was recruited under the study approved by the University of Cape Town Research Ethics Committee (HREC, REF 516/2011). The prospective enrollment of participants in the Peruvian study was approved by the Universidad Peruana Cayetano Heredia Institutional Review Board (SIDISI 65314). The MIMIC study was funded by the Technology Strategy Board UK/Innovate UK and approved by the National Research Ethics Service Committee South Central (Ref 13 SC 0043). University of Southampton Ethics and Research Governance Online approval for transporting samples to the United Kingdom was granted (approval 17758).

## Author contributions

DJGB was involved in the study design, performed the optimization of the proteomic method and conducted the plasma proteome profiling, analyzed and integrated the data and the verification experiments, and wrote the majority of the manuscript. CH White wrote the R scripts used to normalize raw peptide intensities, calculate protein expressions, and perform limma analysis. NFW recruited the South African cohort and provided clinical annotation. MT recruited the MIMIC cohort and provided clinical annotation. HFS was involved in the experiments of verification using ELISA and Luminex. CUG recruited the Peruvian clinical cohort and provided clinical annotation. AM and JA provided expertise in the plasma proteomic protocol. AFV provided expert insight on the bioinformatic analysis and the R scripts for WGCNA and ComBat. MKB was involved in the validation experiments. RJW, SMJ, and BGM assisted with recruitment of patients to the cohorts. LBT assisted in the Luminex analysis. CH Woelk was involved in the study design and provided expertise on the bioinformatic pipeline design. SDG was involved in the study design, provided expertise and advice on the plasma proteomics method, and contributed to the manuscript writing process. PE was involved with the study design, secured funding, and contributed to manuscript writing and editing.

## Supplementary Material

Supplemental data

Supplemental Tables 1-7

## Version 1 08/11/2020

In-Press Preview

## Version 2 09/17/2020

Electronic publication
