# Supplementary material for: Comprehensive plasma proteomic profiling reveals biomarkers for active tuberculosis
Source: JCI Insight. 2020 Sep 17;5(18):e137427. doi: 10.1172/jci.insight.137427 (PMC7526553; doi:10.1172/jci.insight.137427)
Supplement: Supplemental data [file jciinsight-5-137427-s044.pdf]

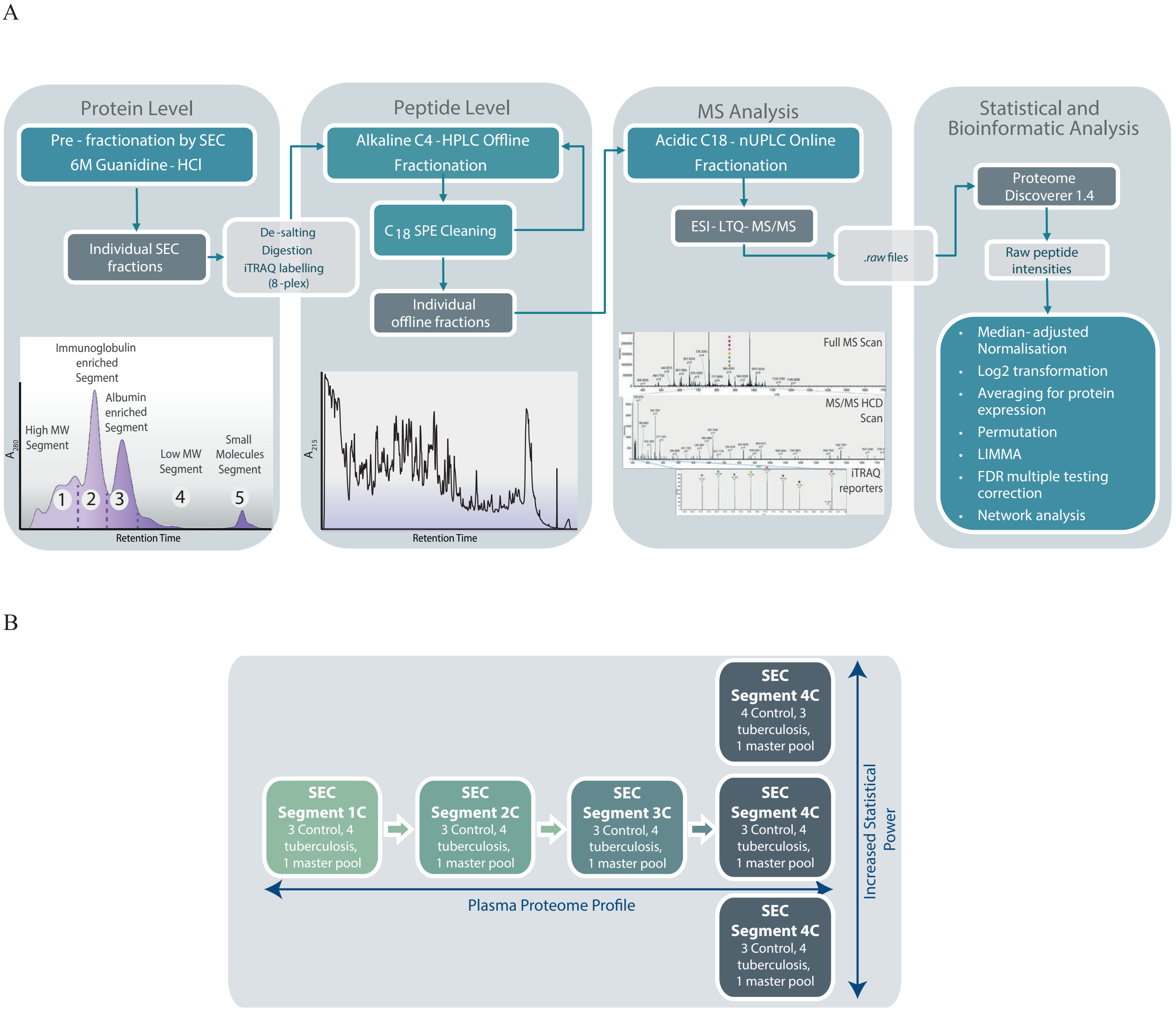

Fig. S1. Experimental strategy for plasma biomarker discovery
(A) The discovery stage involved six 8-plex iTRAQ sets. First, the plasma proteome was profiled across all 4 segments in four TB and three control cases; the remaining tag was used to label the master pool. Next, the sample size was increased for the most informative segment, which involved two further iTRAQ experiments analysing segment 4, thereby reducing the false positive rate for discovery. **(B)** Identification and quantification of plasma proteins was performed using our quantitative multidimensional approach. Schematic depiction of the series of fractionation steps at both protein and peptide level, and summary of the bioinformatics pipeline.

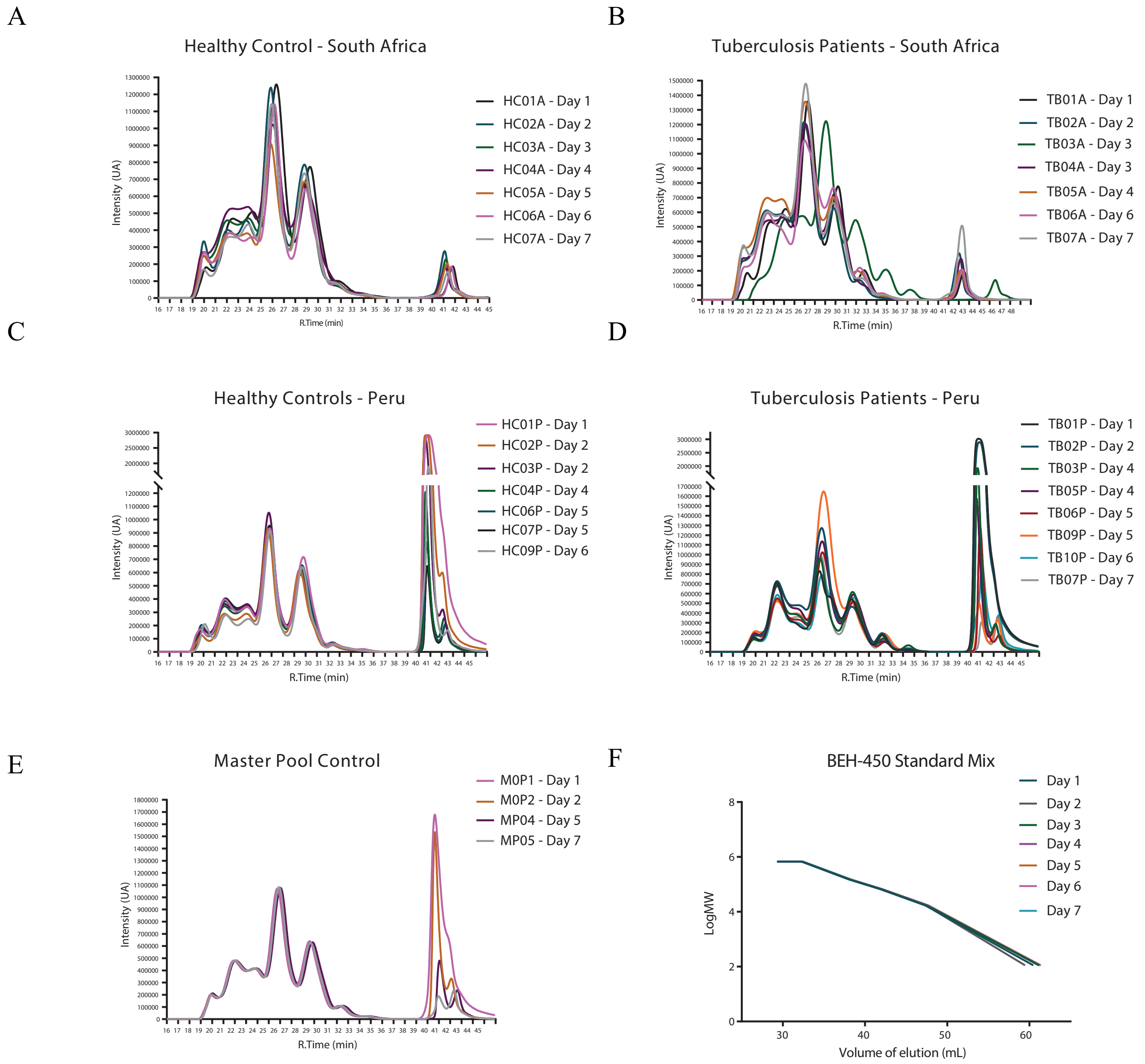

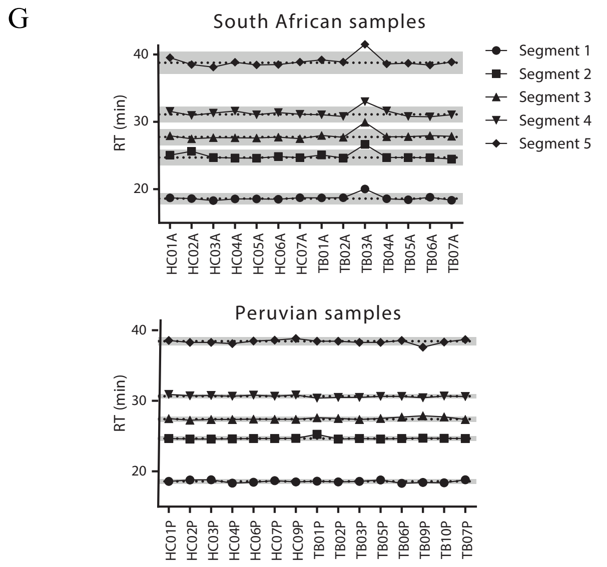

H

SET	Sample ID	Origin	MS Profiled segment
A	TB03A	South Africa	1, 2, 3, 4
	TB04A	South Africa	1, 2, 3, 4
	TB10P	Peru	1, 2, 3, 4
	TB01P	Peru	1, 2, 3, 4
	HC03A	South Africa	1, 2, 3, 4
	HC04A	South Africa	1, 2, 3, 4
	HC04P	Peru	1, 2, 3, 4
B	TB01A	South Africa	4
	TB06A	South Africa	4
	TB03P	Peru	4
	HC02A	South Africa	4
	HC07A	South Africa	4
	HC02P	Peru	4
	HC03P	Peru	4
C	TB05A	South Africa	4
	TB02A	South Africa	4
	TB02P	Peru	4
	TB05P	Peru	4
	HC05A	South Africa	4
	HC01P	Peru	4
	HC07P	Peru	4

Fig. S2. HP-SEC pre-fractionation of plasma samples.
Isocratic chromatographic traces of plasma samples. Intensity was evaluated at 280nm over 45 minutes. Separation was conducted at 1.5mL/min and 45°C. Overlapping of SEC chromatographic traces of **(A)** control samples from South Africa; **(B)** TB samples from South Africa. A single sample had delayed kinetics and segment capture was adjusted accordingly. **(C)** Control samples from Peru. **(D)** TB samples from Peru. **(E)** Master pool samples separation. **(F)** The BEH450 test mix containing six standard proteins with molecular weights ranging from 1.4×10^6Da to 112Da was separated on each day of analysis to evaluate the separation performance and calibration curves are presented (normalised elution volume to void volume V/V_0). **(G)** Collection time-points of SEC segments are plotted for each sample from the South African and Peruvian samples. Grey indicates 2SD. **(H)** Sample classification by analysis set and origin. Segments MS profiled are indicated for each sample.

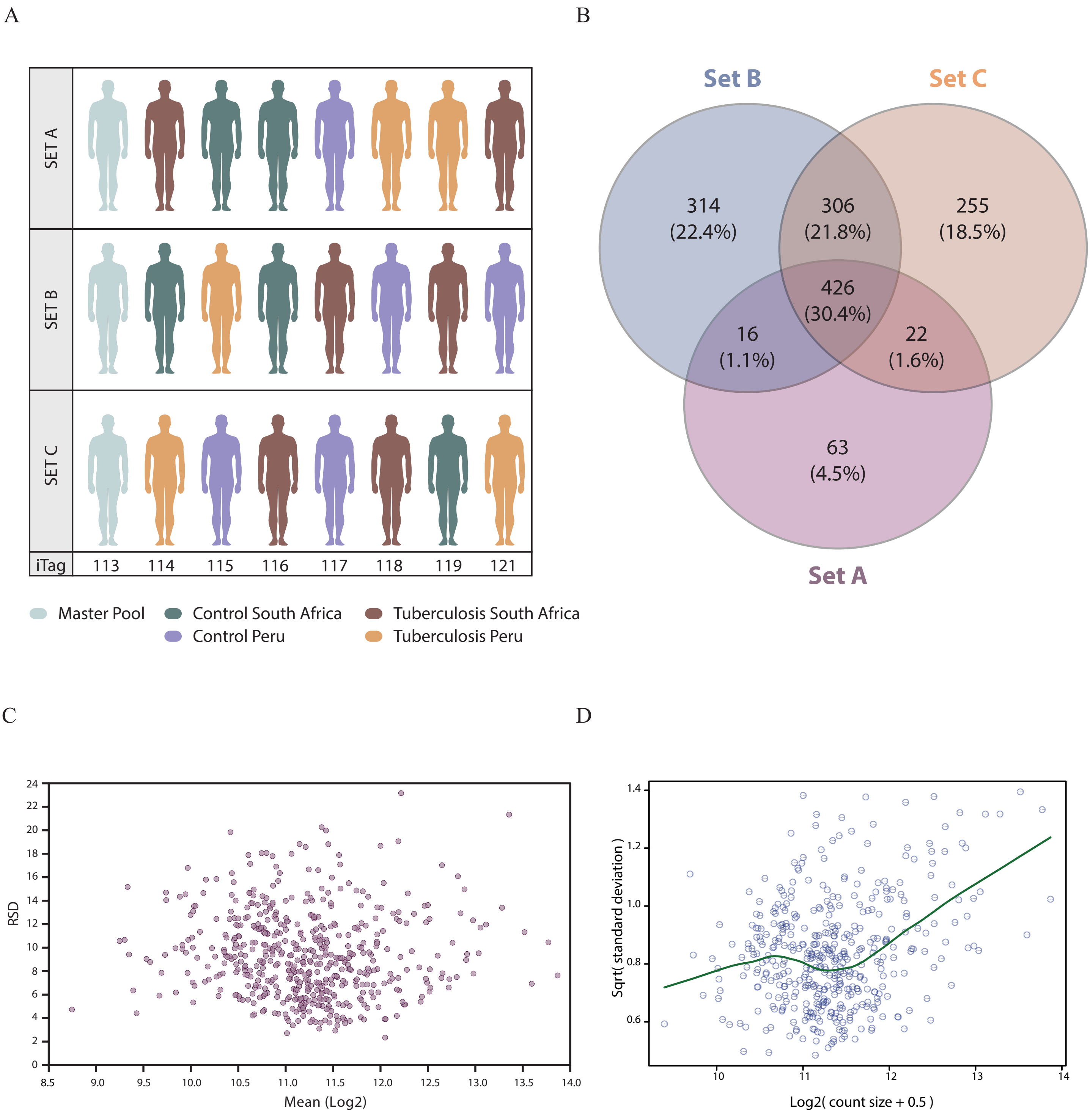

Fig. S3. iTRAQ experiments for in depth profiling of HP-SEC segment 4.

(A) Plasma sample allocation for three iTRAQ 8-plex experiments. Control and tuberculosis samples from Peruvian and South African ethnicities were randomised across three experiments. Tag 113 was assigned to the master pool for variability control between the 8-plex experiments. **(B)** Comparison of fully quantified proteins across sets A, B and C. Evaluation of protein expression variation on common proteins measured across sets A, B and C. **(C)** Scatter plot of the log2 protein relative expression vs. relative standard deviation (RSD). RSD was >25. **(D)** Mean-variance trend plot generated with the function voom from the R package limma. Relative log2 protein expression mean and variances are represented as points with locally weighted regression (LOWESS) trend in green.

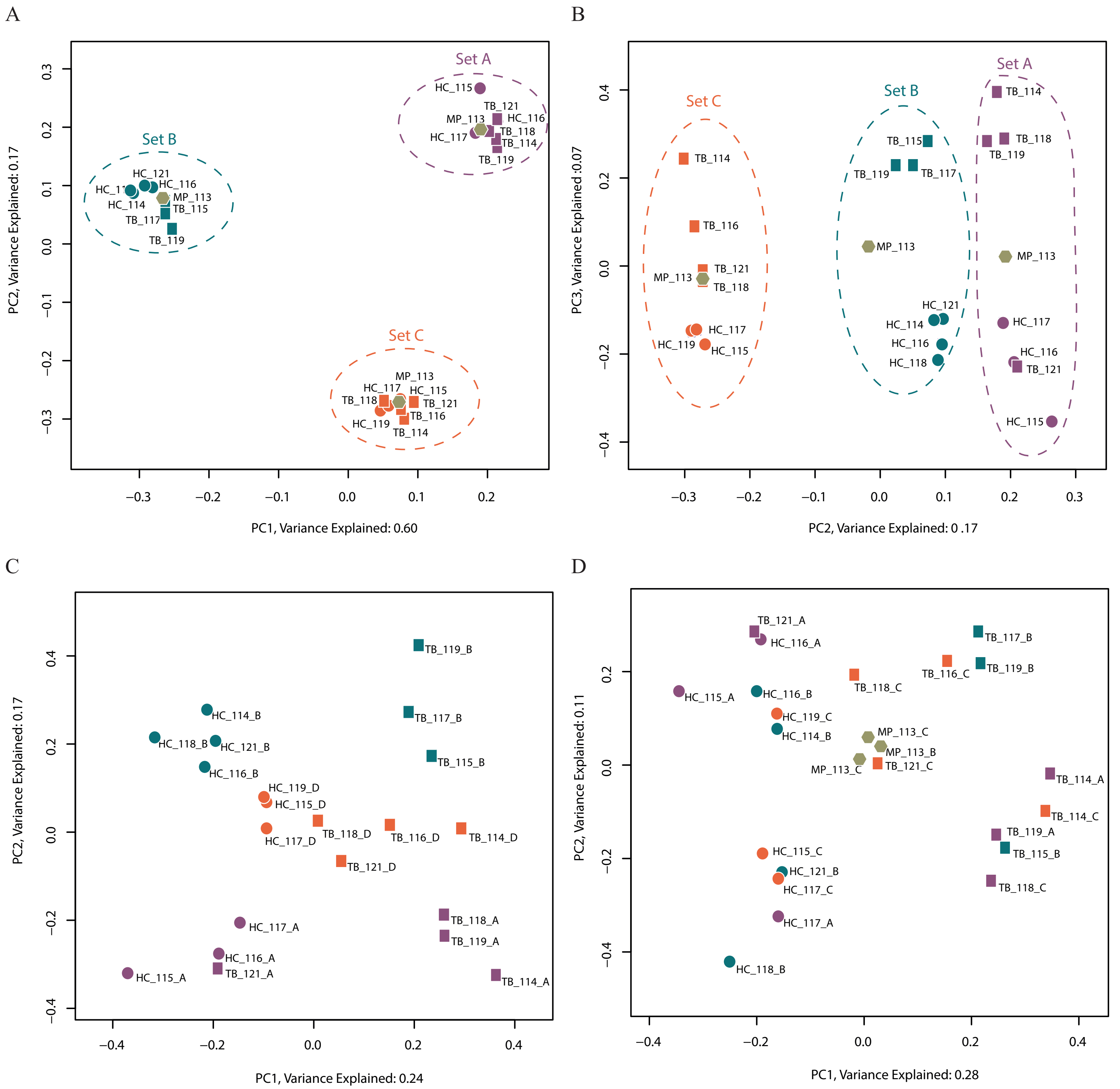

Fig. S4. Batch effect correction across multiple iTRAQ experiments.
Distribution of the relative protein expression of common quantified proteins in HP-SEC segment 4 from sets A, B and C analysed by PCA. **(A)** PC1 and PC2. **(B)** PC2 and PC3. Batch effects were then corrected to combine the datasets and increase statistical power. **(C)** Normalisation to the master pool. **(D)** ComBat batch effect correction, demonstrating the optimal normalisation. Batch A is indicated in purple, batch B in blue and batch C in orange.

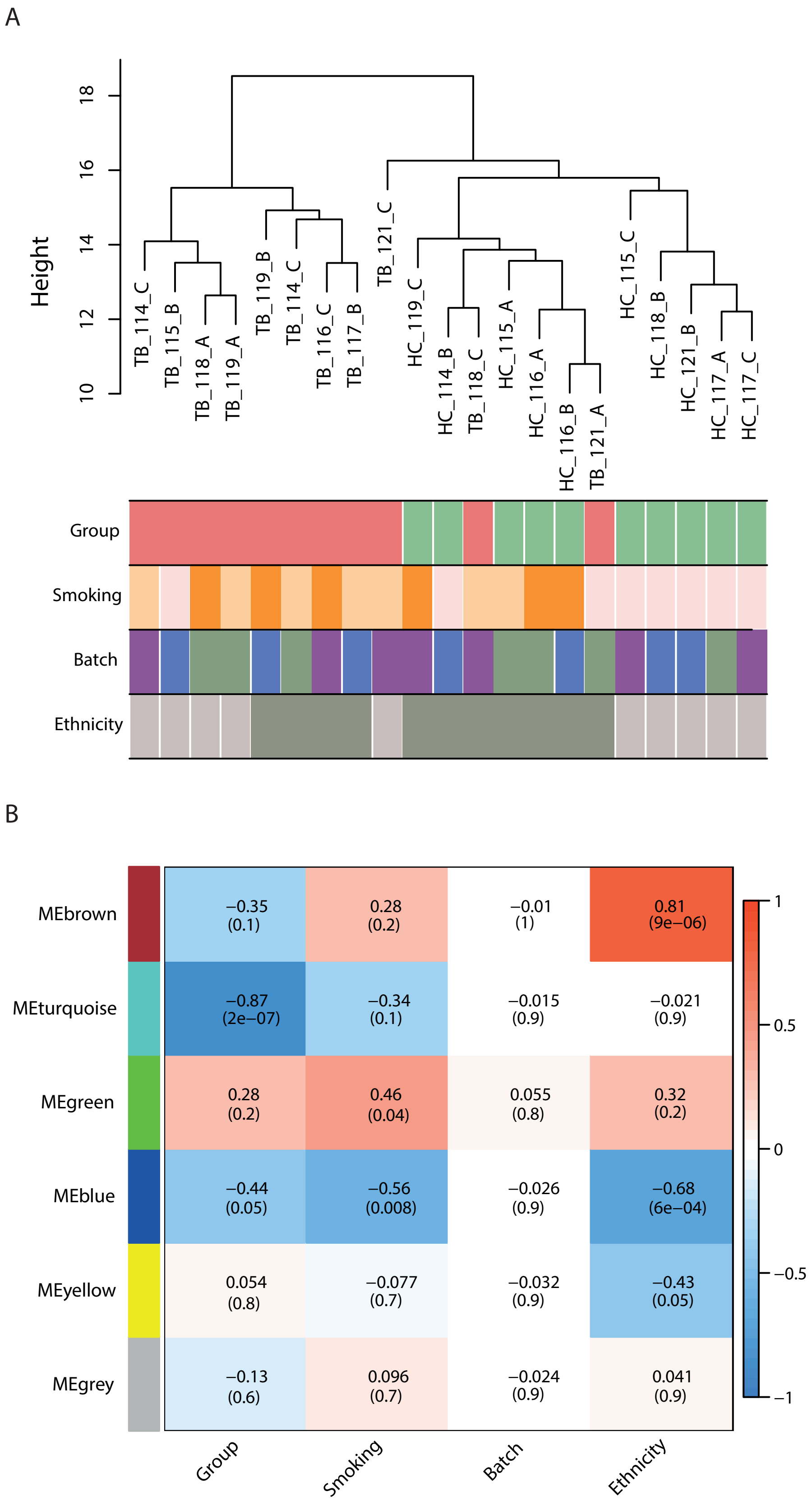

Fig. S5. Evaluation of potential confounding variables in segment 4 proteomic profile.
Group, smoking status, ethnicity and batch were evaluated as possible confounders in the data. **(A)** First, a linkage hierarchical clustering dendrogram were generated using Eclidean distance and variables were colour coded. **(B)** Heatmap of correlations between modules and studied variables. Colours corresponds to correlations. Red indicates positive correlation and blue negative correlation. Correlation scores are labelled and *p* values of correlation are shown in brackets. The module turquoise was strongly correlated to the disease status and the module brown to ethnicity.

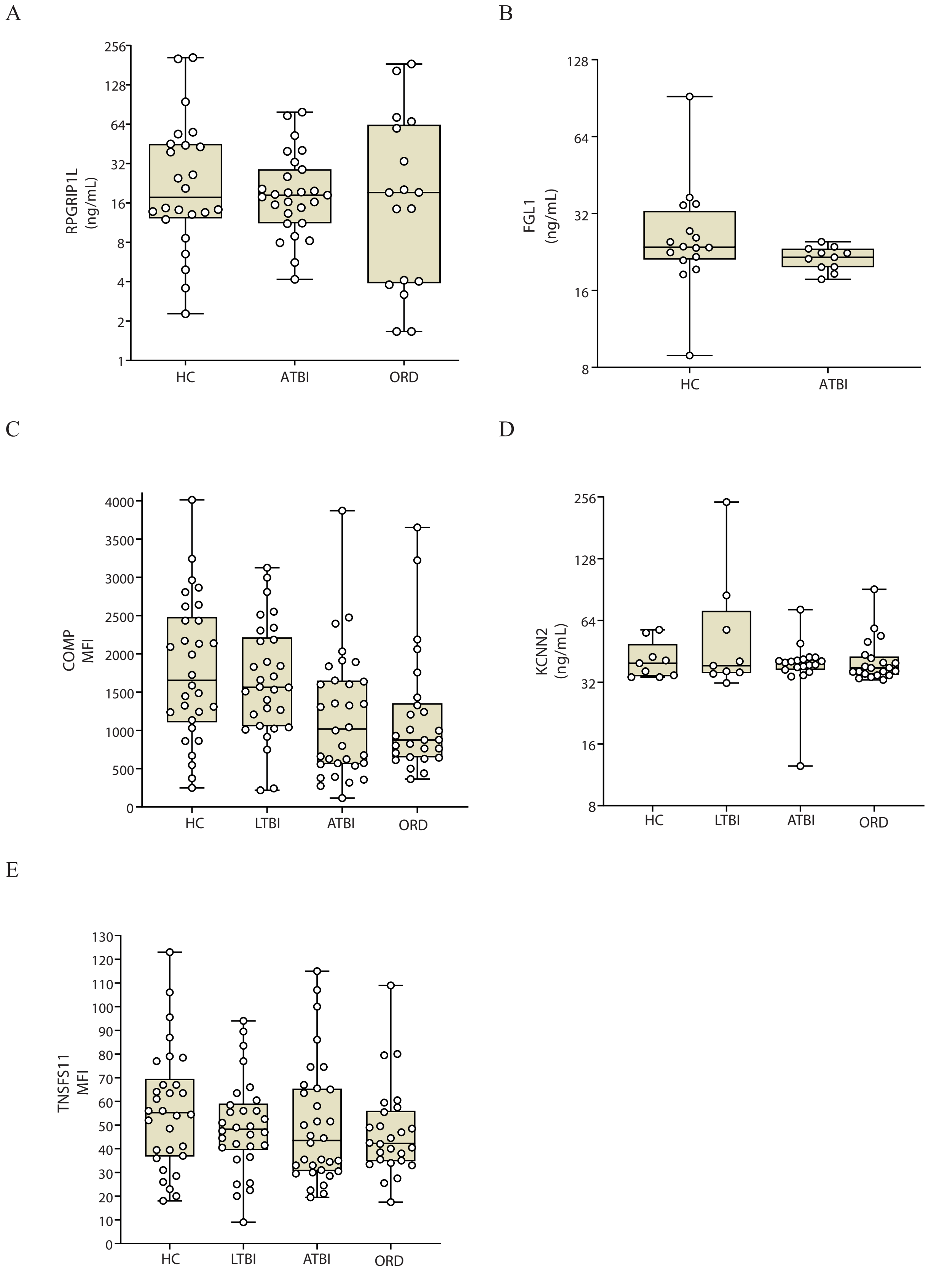

Fig. S6. Candidates of verification subset: Proteins that showed no significant differences
Five candidates showed no significant differences between controls and pulmonary tuberculosis patients, selected on the initial full proteome analysis of 3 controls and four TB patients. Analytes were measured by ELISA or luminex on a subset of plasma samples from the South African or MIMIC verification cohorts **(A)** RPGRIP1L on South African cohort. **(B)** FGL1 on South African cohort. **(C)** COMP on MIMIC. **(D)** KCNN2 on MIMIC. **(E)** TNSFS11 on MIMIC. Box and whisker plots displaying median and minimum to maximum values are shown. Differences were calculated by the Kruskal-Wallis test and Dunn's multiple comparison test. No significant differences were observed between groups. HC: Healthy controls, LTBI: Latent TB infection, PTBI: Pulmonary TB infection and ORD: Other respiratory diseases.

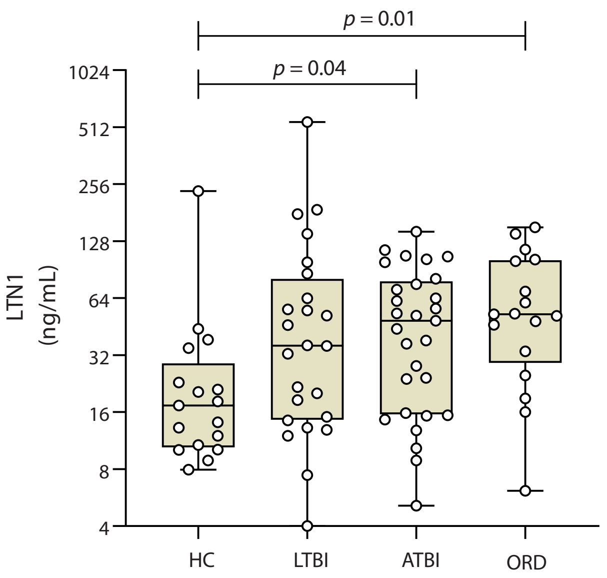

Fig. S7. LTN1 abundance is significantly increased in TB.

LTN1 (E3 ubiquitin-protein ligase listerin) showed significant differences between controls and pulmonary tuberculosis patients when measured by ELISA on a subset of plasma samples from the MIMIC verification cohort. Box and whisker plot displaying median and minimum to maximum values. Expression considered significant when *p-value <0.05* and calculated by the Kruskal-Wallis test and Dunn's multiple comparison test. HC: Healthy controls, LTBI: Latent TB infection, PTBI: Pulmonary TB infection and ORD: Other respiratory diseases.

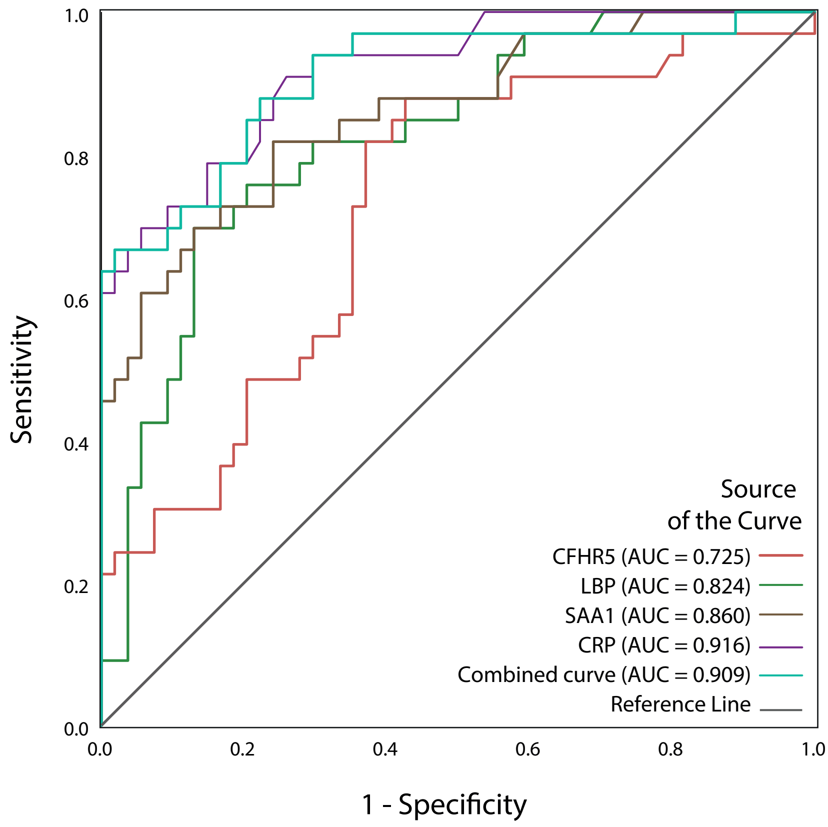

Fig. S8. ROC curve comparing TB patients vs. controls in South African cohort.
ROC curves were generated after binary logistic regression using SPSS v.25, calculated for individual and combined analytes. ROC curve for TB infection vs. control HIV uninfected individuals shows similar differentiation by CRP (AUC = 0.916) and the combined analytes (AUC = 0.909).
